# Non-invasive Multimodality Cardiovascular Imaging of the Right Heart and Pulmonary Circulation in Pulmonary Hypertension

**DOI:** 10.3389/fcvm.2019.00024

**Published:** 2019-03-14

**Authors:** David J. Hur, Lissa Sugeng

**Affiliations:** ^1^Section of Cardiovascular Medicine, Department of Internal Medicine, Yale School of Medicine, New Haven, CT, United States; ^2^Division of Cardiology, Department of Medicine, Veterans Affairs Connecticut Healthcare System, West Haven, CT, United States; ^3^Echocardiography Laboratory, Yale New Haven Hospital, New Haven, CT, United States

**Keywords:** pulmonary hypertension, right heart, non-invasive imaging, multimodality, echocardiography, nuclear cardiology, computed tomography, magnetic resonance

## Abstract

Pulmonary hypertension (PH) is defined as resting mean pulmonary arterial pressure (mPAP) ≥25 millimeters of mercury (mmHg) via right heart (RH) catheterization (RHC), where increased afterload in the pulmonary arterial vasculature leads to alterations in RH structure and function. Mortality rates have remained high despite therapy, however non-invasive imaging holds the potential to expedite diagnosis and lead to earlier initiation of treatment, with the hope of improving prognosis. While historically the right ventricle (RV) had been considered a passive chamber with minimal role in the overall function of the heart, in recent years in the evaluation of PH and RH failure the anatomical and functional assessment of the RV has received increased attention regarding its performance and its relationship to other structures in the RH-pulmonary circulation. Today, the RV is the key determinant of patient survival. This review provides an overview and summary of non-invasive imaging methods to assess RV structure, function, flow, and tissue characterization in the setting of imaging's contribution to the diagnostic, severity stratification, prognostic risk, response of treatment management, and disease surveillance implications of PH's impact on RH dysfunction and clinical RH failure.

## Introduction

**Figure 1:**
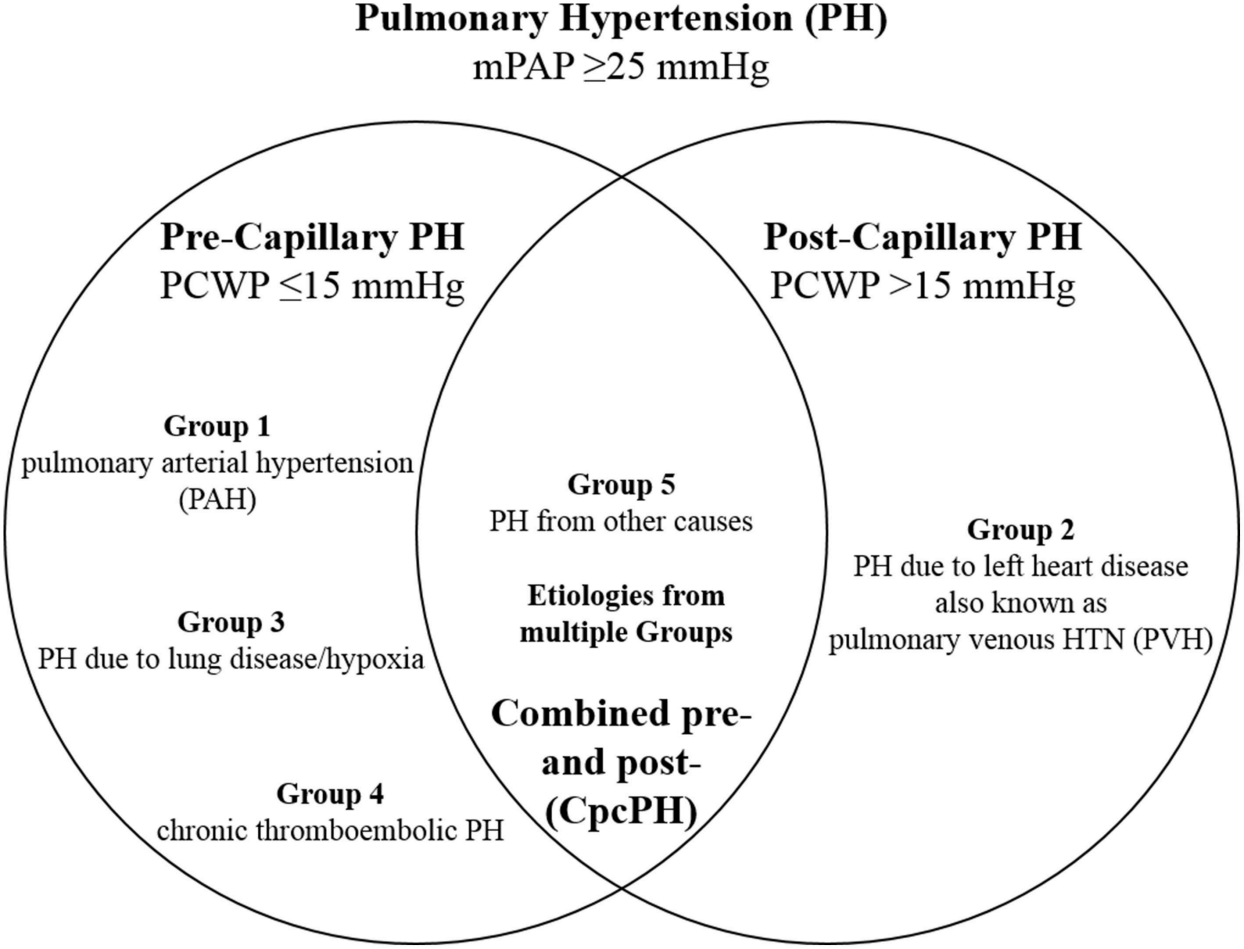
Hemodynamic definitions of pulmonary hypertension (PH) and clinical group classification. Combined pre- and post-capillary pulmonary hypertension (CpcPH) is characterized by pulmonary venous hypertension (PVH), in addition to trans-pulmonary gradient >7 mmHg or pulmonary vascular resistance >3 Wood units. mPAP, mean pulmonary arterial pressure; PCWP, pulmonary capillary wedge pressure. Adapted from Dellegrottaglie et al. (4).

Pulmonary hypertension (PH) is a progressive, potentially life-threatening condition resulting from a variety of causes, defined as an invasively measured resting mean pulmonary arterial pressure (mPAP) ≥25 mmHg (1). The resultant increased afterload in the pulmonary circulation as a result of extensive proliferation and remodeling in the pulmonary artery (PA) vasculature (2) leads to alterations in right heart (RH) structure and function. According to the World Symposium on Pulmonary Hypertension (3), five groups have been defined (Figure 1): group 1 as pulmonary arterial hypertension (PAH), group 2 as PH due to left heart (LH) disease, group 3 as PH due to lung disease and/or hypoxia, group 4 as chronic thromboembolic pulmonary hypertension (CTEPH), and group 5 as PH from other causes; PH can be characterized further by involvement at the pre-capillary and/or post-capillary level. The assessment of PH is based on a comprehensive appraisal of the clinical history, physical examination, and diagnostic studies. However, diagnosis of PH and its subsequent treatment is frequently delayed, as clinical symptoms often overlap with common diseases of the cardiovascular (CV) and respiratory systems (5). The result of often determining PH late in the disease course is that mortality rates have remained high despite therapy (6).

Goals of non-invasive imaging in PH have been focused on helping to establish the diagnosis sooner and identify contributing disease group(s). The need for earlier diagnosis and intervention has been supported, for example, by the finding that PAH patients in World Health Organization functional class (WHO FC) I or II have better long-term survival than those with more severe impairment (WHO FC III or IV) (7, 8). The hope of expediting correct group classification of etiology is that delineating the underlying cause will impact on prognosis and management (3), and ultimately coming to an early, accurate diagnosis of PH may allow for earlier treatment and the chance for improved outcomes (9, 10). Historically, the 6-min walk test has been used to monitor the effects of treatment on PH (11). However, in a meta-analysis involving more than 20 clinical trials showed that the 6-min walk test was not predictive of outcomes (12). Thus, there has been an ongoing push to elucidate other quantitative and imaging-based markers of PH severity that can be used to document response to therapy, both for use in clinical trials and ultimately with the aim for real-world practice.

### Approach to PH Evaluation Using Imaging

**Figure 2:**
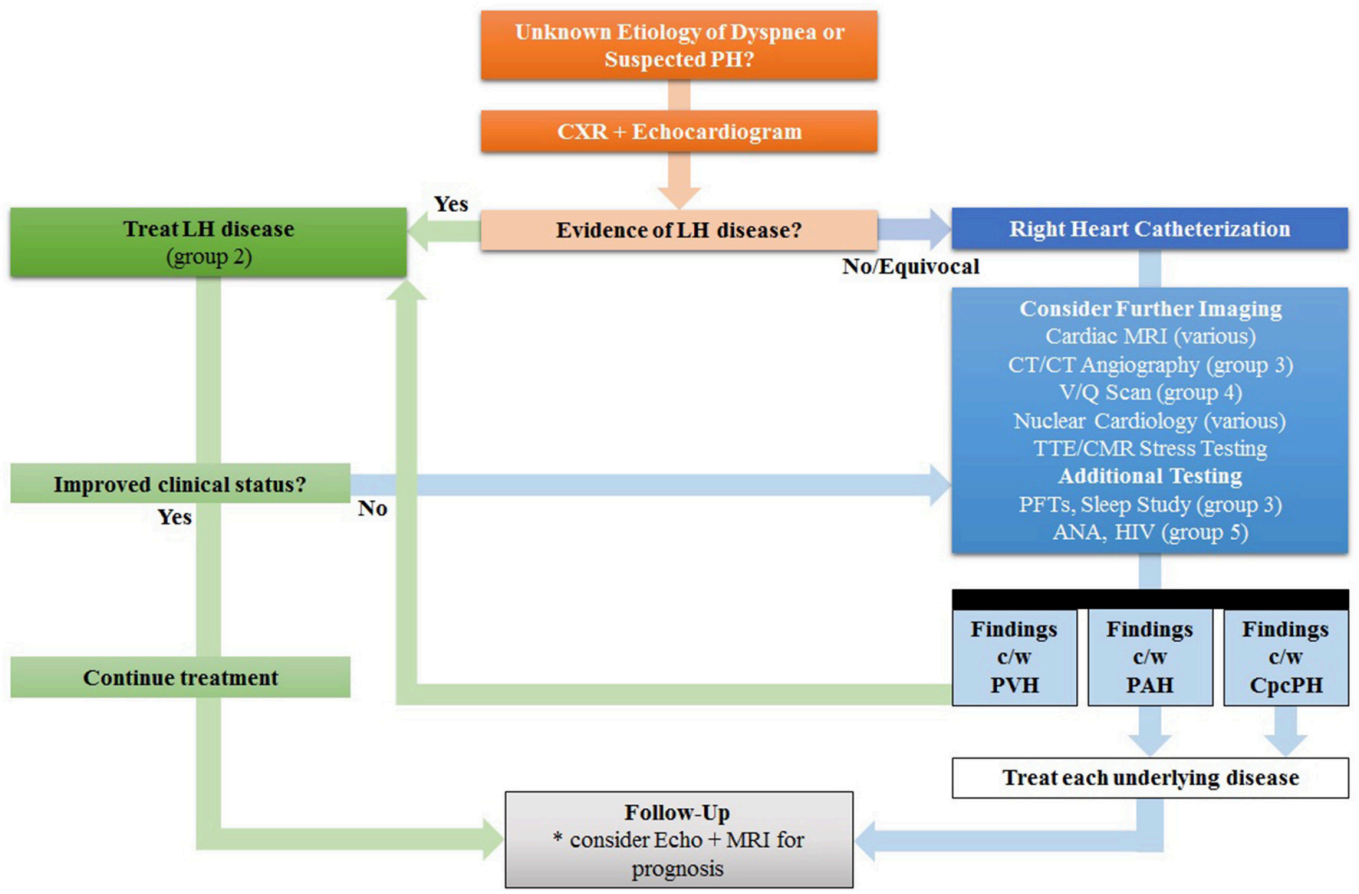
Proposed Step-Wise Approach In Lieu of Traditional “Shotgun” Approach for the evaluation of Pulmonary Hypertension (PH). Since many patients with PH in the developed world have left heart (LH) disease, the echocardiogram is the preferred test for initial cardiac work-up; if findings are consistent with pulmonary venous hypertension (PVH), treatment for left heart disease is recommended first before automatically proceeding to other tests, such as invasive hemodynamic testing. Other signs of LH disease include reduced left ventricular ejection fraction, aortic valve disease, and mitral valve disease. Treatment of LH disease is dependent on the underlying etiology, but in group 2 PH many patients will improve with diuresis and lowering of systemic blood pressure with vasodilators. Further non-invasive imaging or invasive catheterization can be considered as second-line options. MRI can be considered for prognosis and follow-up. ANA, antinuclear antibody, CpcPH, combined pre- and post-capillary pulmonary hypertension; CT, computed tomography; HIV, human immunodeficiency virus; LH, left heart; MRI, magnetic resonance imaging; PAH, pulmonary arterial hypertension; PFTs, pulmonary function tests; V/Q, ventilation/perfusion. Adapted from Freed et al. (18).

Right ventricular failure is frequently the cause of death in PH (13, 14). Thus, the historical view that the right ventricle (RV) being a passive chamber with minimal role in the overall function of the heart has changed in recent years, particularly in the evaluation of PH. This has given rise to increasing attention and focus on the RV regarding its performance and relationship to other structures in the RH-pulmonary circulation, including the PA, tricuspid valve (TV), and right atrium (RA). The shape of the RV is more complex than that of the left ventricle (LV). The RV has superficial circumferential muscle fibers responsible for an inward bellows movement of the free wall contracting toward the rigid septum, as well as inner longitudinal fibers that result in the base-to-apex contraction movement (15). Compared to the LV, the base-to-apex shortening provides a greater contribution to RV emptying (16). RV function is a reflection of mechanical coupling between the RV and PA (17). Due to its shape and location, as well as the load dependence of the ejection fraction (EF), accurate evaluation of RV function remains challenging. The various imaging modalities, with differing degrees of sensitivity and specificity, provide incremental, often complementary information. This review provides an overview and summary of the current non-invasive imaging methods to assess RV structure, function, flow, and tissue characterization. Emerging evidence and established guidelines are discussed in the setting of imaging's contribution to diagnosis, severity stratification, prognostic risk, treatment management response, and disease surveillance of PH and its impact on RH dysfunction and clinical RH failure. The utility, advantages, and limitations of the imaging modalities that are used to evaluate the RV in patients with suspected or known PH will be discussed. Table 1 summarizes the strengths and limitations of the various imaging techniques. With the aim of value-based decision-making, a judicious step-wise approach algorithm to utilization of imaging in PH evaluation may reduce unnecessary testing and improve the value of care (Figure 2).

**Table 1:** Relative strengths and limitations of non-invasive imaging modalities used in pulmonary hypertension.

	**CXR**	**Echo**	**CMR**	**CT/CTA**	**Radionuclide (including V/Q)**
Initial assessment & diagnosis	+	++	+	+/+	
Evaluation of etiology	++	++	++	++/+++	++
Cardiac Structures/Cardiovasculature	+	++	+++	++/++	++
Pulmonary vasculature	+	+	++	+++/+++	+
Lung parenchyma	+		+	+++/+++	
**LOGISTICAL CONSIDERATIONS**					
Portability		+++			
Patient accessibility/Ease of monitoring		+++	+	++/++	
Quickness of study duration		++	+	+++/+++	
Non-contrast capability		+++	++	+++/+	
Radiation exposure		+++	+++	+/+	
**IMAGING CONSIDERATIONS**					
RV Ejection fraction		++ (3DE)	+++	++	
Regional wall motion		++ (3DE)	+++	++	
3D Volumes		++ (3DE)	+++	+++	
RV Morphology		++	+++	+++	
Tissue characterization			+++	++	
Pulmonary artery pressures		+++	++		
Strengths	Widely available, low cost; ability for portable scans	Radiation-free; readily available; ability for portable scans; good for initial PH eval	Radiation-free; low interobserver variability; tissue characterization	Short scan time; utility in evaluation of etiologies of PH	Good for initial CTEPH eval
Limitations	Disease severity does not directly correlate with changes seen on plain film	Need for downstream testing due to reduced image quality related to body habitus	May cause claustrophobia; cannot be used in those with certain ferromagnetic implants; requires breath holding; long scan time; reduced image quality due to motion artifacts	Radiation risk; contraindicated in pregnancy; limited hemodynamic eval	Radiation risk; contraindicated in pregnancy; non-specific or non-diagnostic findings; need for downstream testing
Comments	Part of initial work-up for shortness of breath	Imaging study of choice for group 2 PH; can assess systolic and diastolic function, valvular heart disease, congenital heart disease/shunts	Plays role in eval for myocardial scar/fibrosis; can provide complementary information to echo regarding anatomy and RV function	Imaging study of choice for groups 3 and 5 PH; can assess lung diseases (emphysema, ILD) and multifactorial diseases (sarcoidosis, fibrosing mediastinitis); can provide complementary role to echo regarding anatomy	Imaging study of choice for group 4 PH

*+, limited utility; ++, moderate utility; +++, high utility; 3DE, three-dimensional echocardiography; CMR, cardiac magnetic resonance; CT, computed tomography; CTA, computed tomography angiography; CTEPH, chronic thromboembolic pulmonary hypertension; CXR, chest radiography; ILD, interstitial lung disease; PH, pulmonary hypertension; RV, right ventricular; V/Q, ventilation/perfusion scan*.

## Initial Evaluation of the Patient Referred for Previously Unexplained Dyspnea or Suspected PH

The initial evaluation of individuals with unelucidated causes of shortness of breath should involve chest radiography (CXR) and transthoracic echocardiography (TTE).

### Chest Radiography

**Figure 3:**
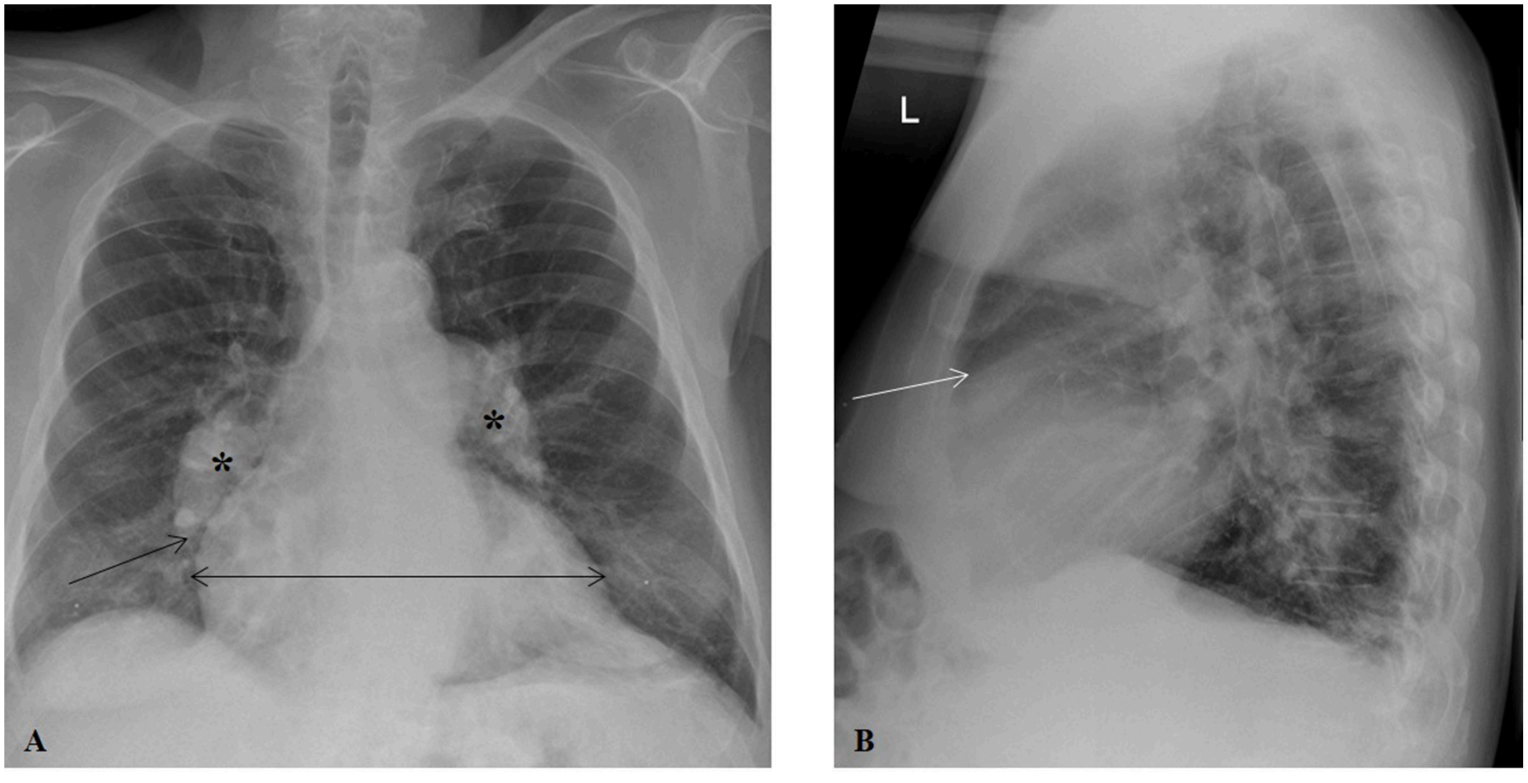
Chest radiography in a patient with groups 2 and 3 pulmonary hypertension. **(A)** Posteroanterior projection illustrating prominent, dilated central pulmonary arteries (asterisks), cardiomegaly (horizontal line), and rapid tapering (pruning) of right pulmonary artery (arrow). **(B)** Lateral projection showing a decrease in the retrosternal air space (arrow).

CXR (Figure 3) is usually part of the initial or baseline evaluation of patients with unexplained shortness of breath or other symptoms that could be due to PH. It remains widely used due to availability and relatively low cost. It can depict features of PH that are indicative of the underlying cause, such as lung abnormalities in patients with PAH secondary to systemic sclerosis (group 1), PH secondary to lung parenchymal diseases, such as emphysema or interstitial lung disease (group 3), or PH secondary to CTEPH (group 4) in the setting of sign(s) of pulmonary embolism (Westermark's sign, a wedge-shaped shadow indicating pulmonary infarction, or Hampton's hump, a wedge-shaped opacification secondary to pulmonary hemorrhage).

Chest plain X-ray films can uncover significant pulmonary vascular abnormalities by showing vessel displacement and changes in vascularity of the lungs (19). While a normal CXR does not necessarily exclude PH given that it is usually not sensitive for the identification of patients with mild to moderate PH (20), it is commonly abnormal in established disease and can detect the typical changes seen in the PAs and RH in those with longstanding, severe PH (21). These classic, late findings of PH on CXR include characteristic dilation of the main and central PAs (22), while the branch PAs and peripheral blood vessels become abruptly tapered, resulting in a pruning appearance (23). Calcific, atherosclerotic changes may be seen along the central PAs (24), and PA-pulmonary vein ratio may be altered; increased ratio suggests arterial PH, whereas decreased ratio indicates venous PH (25). The CXR can show signs of cardiac involvement as well. The RH may appear enlarged, as suggested by cardiomegaly and prominence of the right border, a result of RA enlargement, on posteroanterior projection and decreased retrosternal air space and obliteration of clear space in the lateral projection, a result of RV dilatation.

### Transthoracic Echocardiography

**Figure 4:**
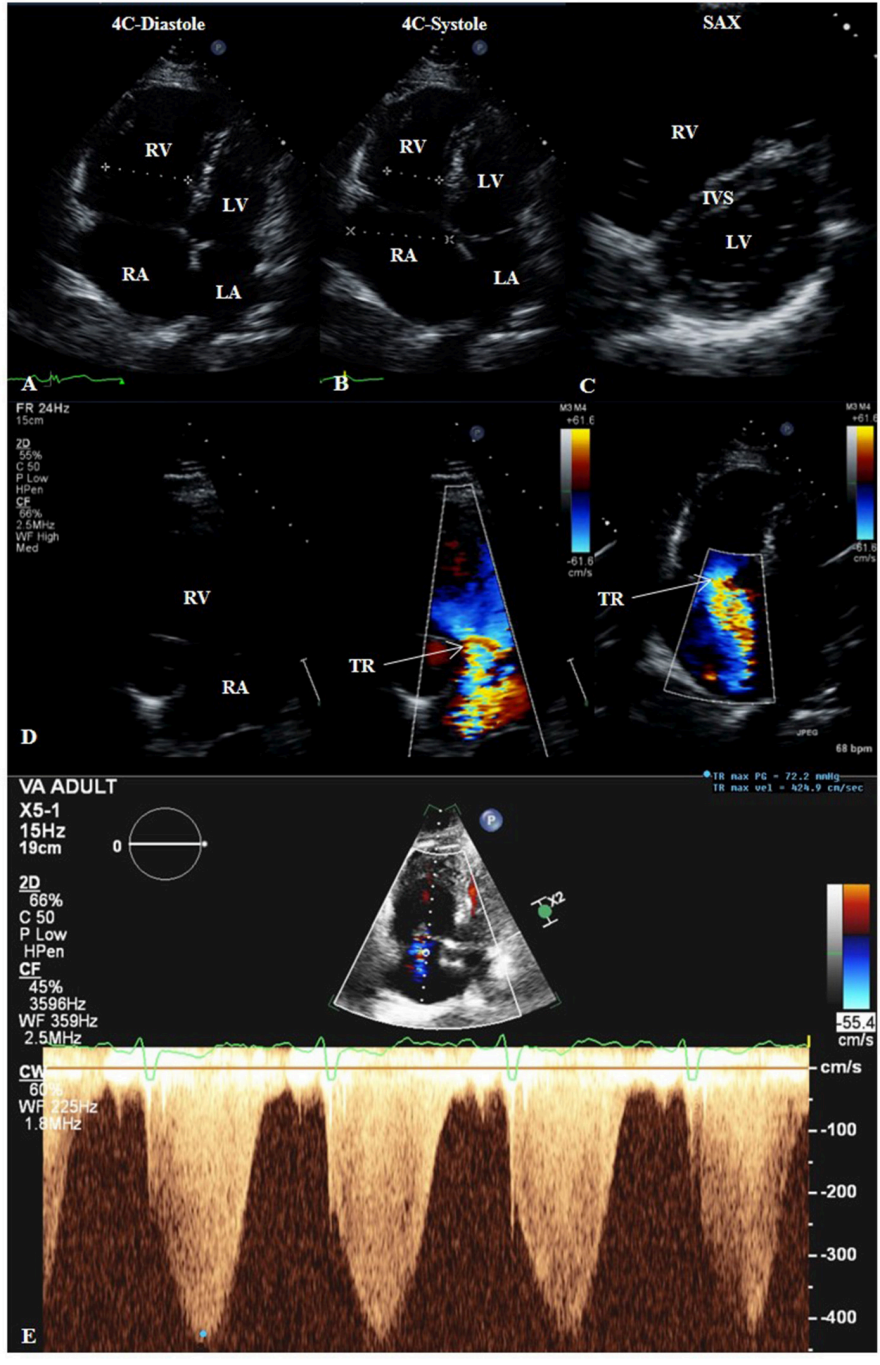
Transthoracic echocardiogram (TTE) of a patient with groups 2 and 3 pulmonary hypertension. 2D TTE in the apical four-chamber (4C) view in diastole **(A)** and systole **(B)** showing right heart dilatation. 2D TTE in the short-axis (SAX) view **(C)** showing flattening of the interventricular septum compatible with the right heart findings. **(D)** Color-compare TTE in the right ventricular inflow view and color Doppler TTE in the 4C view showing severe tricuspid regurgitation. **(E)** Continuous-wave Doppler TTE showing maximum trans-tricuspid valvular regurgitant jet velocity of 4.25 m/s, correlating to estimated pulmonary artery systolic pressure of 72 mmHg plus right atrial pressure. 4C, four-chamber; IVS, interventricular septum; LA, left atrium; LV, left ventricle; RA, right atrium; RV, right ventricle; SAX, short-axis; TR, tricuspid regurgitation.

TTE has a leading role in the PH guidelines, recommended as a first-line non-invasive imaging test to be performed as part of the initial workup for suspected PH (26). It is safe, widely available, reproducible, and relatively inexpensive (27). Echo is an essential non-invasive cardiac imaging screening tool for determining the presence of PH, especially in those where the condition has high probability to be expected (28). TTE's role is vital in the investigation in determining the most common cause of PH in the developed world, group 2 (PH due to LH disease); it may also be useful in patients with family history of PAH, congenital heart disease (CHD), systemic to pulmonary shunts, portal hypertension, or systemic diseases associated with PH (29, 30). The modality can provide two- (2D) and in cases with good image quality three-dimensional (3D) information on several parameters in the evaluation for PH. Echo can assess for chamber (RV and RA) size (Figures 4A–C) (31). RV systolic function can be assessed in a variety of ways, including via tricuspid annular plane systolic excursion (TAPSE), Doppler-derived systolic velocity of TV annulus (S'), fractional area change (FAC), RV myocardial performance (also known as Tei) index (RIMP), RV dP/dt, RVEF, and RV strain (16, 32).

TTE also has the capability to assess hemodynamics, such as estimation of pulmonary artery systolic pressure (PASP) from maximum trans-tricuspid valvular regurgitant jet velocity (TRjetV) as determined from continuous-wave Doppler in addition to right atrial pressure (33). The peak pressure gradient (Δ*P*) of tricuspid valve insufficiency (TR, Figure 4D) is calculated via the modified Bernoulli equation:

$$\begin{array}{l} \Delta P=4\times \;{\text{v}}^{2}, \end{array}$$

where *v* is the Doppler imaging-derived maximal TRjetV in meters per second (m/s) (Figure 4E) (16). The right atrial pressure (RAP) in millimeters of mercury (mmHg) is estimated by quantifying the collapsibility index (34) of the inferior vena cava (IVC), acquired from the subcostal view with the patient in the supine position and measured in the long-axis view 1-2 cm from the IVC-RA junction, along with an inspiratory sniff (31). The following RAP values have been proposed (35): IVC diameter <2.1 cm that collapses >50% with a sniff suggests normal RAP of 3 mmHg (range, 0-5 mmHg), whereas IVC diameter >2.1 cm that collapses <50% with a sniff suggests high RAP of 15 mmHg (range, 10–20 mmHg); in situations in which IVC diameter and collapse do not fit this model, an intermediate value of 8 mmHg (range, 5–10 mm Hg) may be used. PH is considered possible when: (1) TRjetV 2.9–3.4 m/s and PASP 37–50 mmHg, regardless of whether other signs of PH are present; or (2) TRjetV ≤2.8 m/s, PASP ≤36 mmHg, but additional echo variables suggestive of PH are present (33). In their 2015 guidelines for the diagnosis and treatment of PH, the joint task force of the European Society of Cardiology and the European Respiratory Society recommend grading the probability of PH based on TRjetV at rest and the presence of additional echo variables (PA diameter >2.5 cm, early diastolic pulmonary regurgitation velocity >2.2 m/s, flattening of the interventricular septum (LV eccentricity index >1.1 in systole and/or diastole), RV/LV basal diameter ratio >1.0, RV outflow Doppler acceleration time <105 ms and/or mid-systolic notching, RA area (end-systole) >18 cm^2^, IVC >2.1 cm with decreased inspiratory collapse (<50% with a sniff or <20% with quiet inspiration) (26). The probability should be considered high if: (1) TRjetV >3.4 m/s (even with no other signs); or (2) TRjetV ≥2.9 m/s and additional echo variables suggestive of PH are present. The probability should be considered intermediate if: (1) TRjetV 2.9–3.4 m/s with no other signs; or (2) TRjetV ≤2.8 m/s (or insufficiently measurable due to trivial envelope), but accompanied by additional signs of PH. The probability can be considered low if TRjetV ≤2.8 m/s or not measurable, and there are no additional echo variables suggestive of PH. Correlations between echocardiographic Doppler imaging and invasive measurements of PASP are high (36). However, discordance between non-invasive and invasive pressure measurements can occur; causes of inaccurate PA pressure measurement by TTE include poor acoustic windows, Doppler misalignment with the TR jet, interobserver variability in measuring the TRjetV, and breakdown in the assumptions inherent in the modified Bernoulli equation. In the absence of a gradient across the RV outflow tract or pulmonic valve, PASP is equal to RV systolic pressure (RVSP).

Other parameters that can be ascertained by echo include PA diastolic pressure (PADP) and mPAP. PADP can be estimated from the end-diastolic pulmonic regurgitant jet velocity using the continuous-wave Doppler signal of pulmonic insufficiency and modified Bernoulli equation: PADP = 4 × (end-diastolic pulmonic regurgitant jet velocity)^2^ + RAP (16). mPAP can be determined in several ways: mPAP = $\frac{1}{3}$(SPAP) + $\frac{2}{3}$(PADP); or estimated by using pulmonary acceleration time as measured by pulse-wave Doppler of the PA in systole and determined by the following formulas: mPAP = 79 – (0.45 × systolic PA acceleration time) or 90 – (0.62 × systolic PA acceleration time) in those with acceleration time less than 120 ms (37); or correlated to 4 × (early or maximal pulmonic regurgitant jet velocity)^2^ + RAP (38).

Finally, more recently a novel parameter, echocardiographic pulmonary to left atrial ratio (ePLAR) has been described to distinguish between pre-capillary and post-capillary PH (39). ePLAR has been proposed as an echo surrogate for trans-pulmonary gradient and its interaction with LA pressure: ePLAR = TRjetV_max_ / (mitral E/e'); values for normal reference population were 0.30 ± 0.09 m/s; pre-capillary PH patients (ePLAR 0.44 ± 0.22 m/s) had significantly higher values than post-capillary PH patients (ePLAR 0.20 ± 0.11 m/s, *p* < 0.001). Combined pre- and post-capillary PH patients had intermediate values (ePLAR 0.28 ± 0.18 m/s), but remained significantly higher than in those with isolated post-capillary PH (those with post-capillary PH further distinguished from combined by having diastolic pulmonary gradient <7 mmHg; ePLAR 0.18 ± 0.08 m/s, *p* < 0.001).

## Advanced and Adjunct Techniques

If there is evidence of LH disease by CXR and echo, the clinician should proceed to treat the LH disease. However, when there is no overt or equivocal evidence of LH disease, further non-invasive imaging should be considered in conjunction with RHC, which may include cardiovascular magnetic resonance (CMR) imaging, computed tomography (CT), and radionuclide studies.

### Cardiovascular Magnetic Resonance

CMR is a 3D tomographic technique that, due to high definition of the blood pool-myocardium interface and reproducibility of RV parameters (40) has become the non-invasive reference standard for assessing right-sided chamber sizes, morphology, and myocardial mass, as well as transvalvular flow (41). CMR allows for accurate quantification of RV volumes, mass, and EF (15, 42). It is useful in the work-up of cardiomyopathy as it has the potential to detect myocardial inflammation and fibrosis, both ischemic-mediated scar from infarction and non-ischemic scar from infiltrative processes (43). While CMR is expensive, less widely available, and requires operator expertise and high technical demands, including longer scan times, it is safe and does not expose patients to ionizing radiation. Compared to TTE, CMR offers improved spatial resolution not limited by acoustic windows.

CMR scans include cine, phase contrast (PC), and post-contrast sequences. For cine imaging, a four-chamber steady-state free precession (SSFP) cine stack in axial oblique view is typically obtained for the RV. These images are usually obtained at 8-mm slice intervals with a 2-mm inter-slice (25%) gap. Additionally, a short-axis SSFP cine stack can be obtained. Next, PC imaging of the main, right, and left PAs is obtained to assess peak velocities and differential flow to the right and left lungs. Also, valve regurgitation such as TR severity can be quantified either directly or indirectly. Then, contrast-enhanced magnetic resonance angiography (MRA) can be performed. In patients for whom gadolinium-based contrast agent (GBCA) is contraindicated, non-contrast MRA techniques such as navigator (electrogram [ECG] and respiratory)-gated whole-heart SSFP MRA can be used, although this can be suboptimal for the evaluation of distal segmental and subsegmental vasculature. Finally, delayed enhancement (DE) imaging can be obtained 10–15 min after receipt of intravenous (IV) GBCA, permitting visualization of myocardial scar.

#### CMR Techniques for RH Structure, Function, and Tissue Characterization

CMR allows for additional valuable characterization of the RH in patients with PH, providing information on RV size, morphology, volumes, interventricular septum (IVS) shape, and function (44), as well as RV mass (45, 46) and left-right delay in myocardial shortening (47).

**Figure 5:**
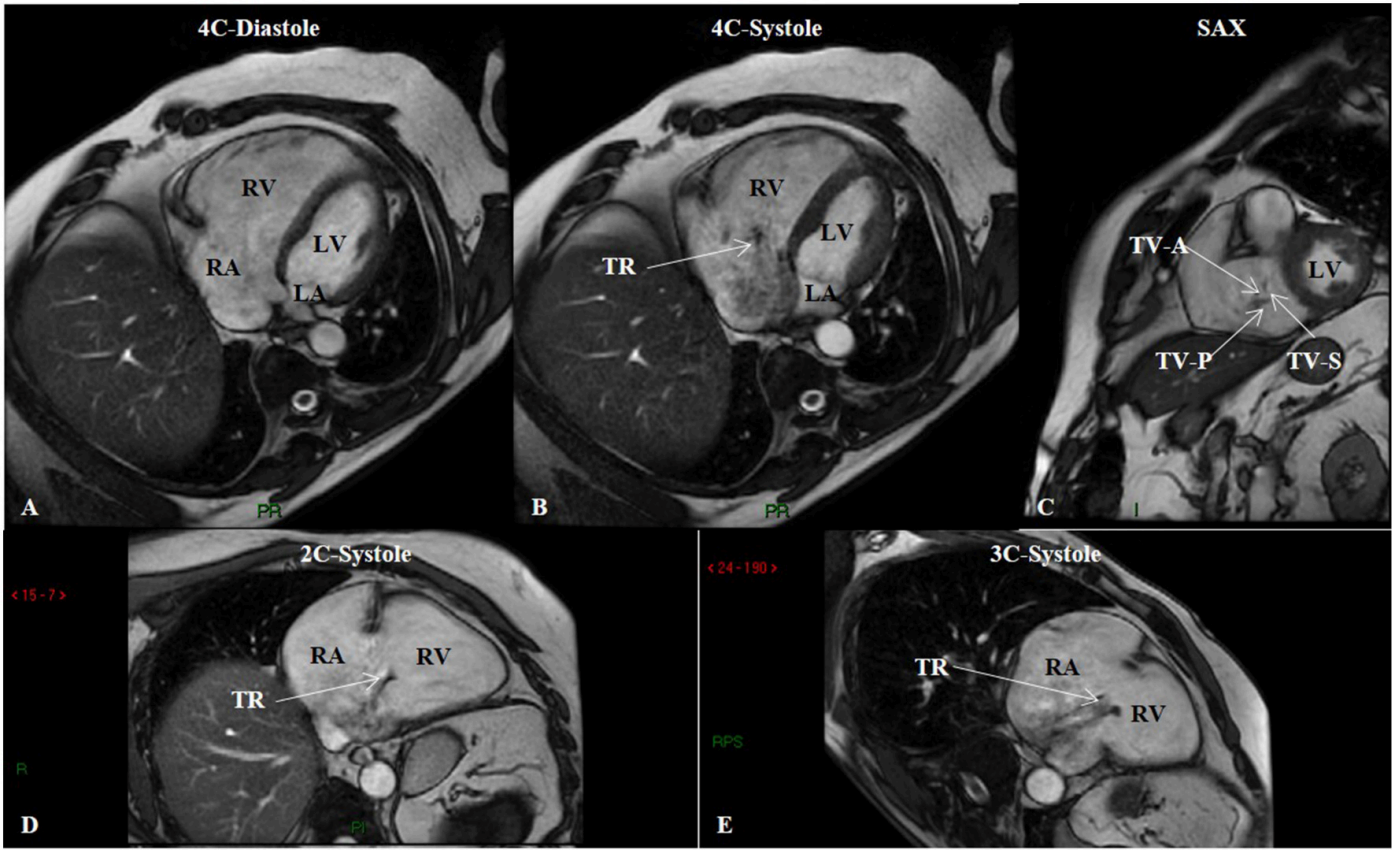
Steady-state free precession (SSFP) cine imaging by cardiac magnetic resonance of a patient with group 3 pulmonary hypertension. SSFP in the four-chamber (4C) orientation at end-diastole **(A)** showing right ventricular enlargement, and at mid-systole **(B)** showing tricuspid regurgitation. **(C)** SSFP in the short-axis (SAX) orientation depicts malcoaptation of the tricuspid valve leaflets. SSFP in the two-chamber (2C) **(D)** and three-chamber (3C) **(E)** orientations in systole showing again tricuspid regurgitation. LA, left atrium; LV, left ventricle; RA, right atrium; RV, right ventricle; TR, tricuspid regurgitation; TV-A, tricuspid valve anterior leaflet; TV-P, tricuspid valve posterior leaflet; TV-S, tricuspid valve septal leaflet.

ECG-gated SSFP cine images can quantify RV chamber size and systolic function (18). RV cardiac output can be estimated by multiplying the determined RV stroke volume (SV) by the heart rate during the image acquisition of the cine stacks. Axial views of the heart chambers reveal a change in RV morphology from its usual crescent shape to a more rounded contour due to concentric hypertrophy, as well as RV dilation. Other abnormalities that can be seen with PH include RV hypertrophy, reduced RVEF, RV wall motion abnormalities, septal abnormalities, TR, and RA enlargement (Figure 5) (48). In individuals with mPAP ≥30 mmHg, a systolic leftward deviation of the IVS had 86% sensitivity and 91% specificity for detecting PH (49). Finally, RV mass is greater in PH compared to healthy controls; a ventricular mass index (RV mass divided by LV mass) >0.6 is suggestive of PH (sensitivity 84% and specificity 71%) (40).

**Figure 6:**
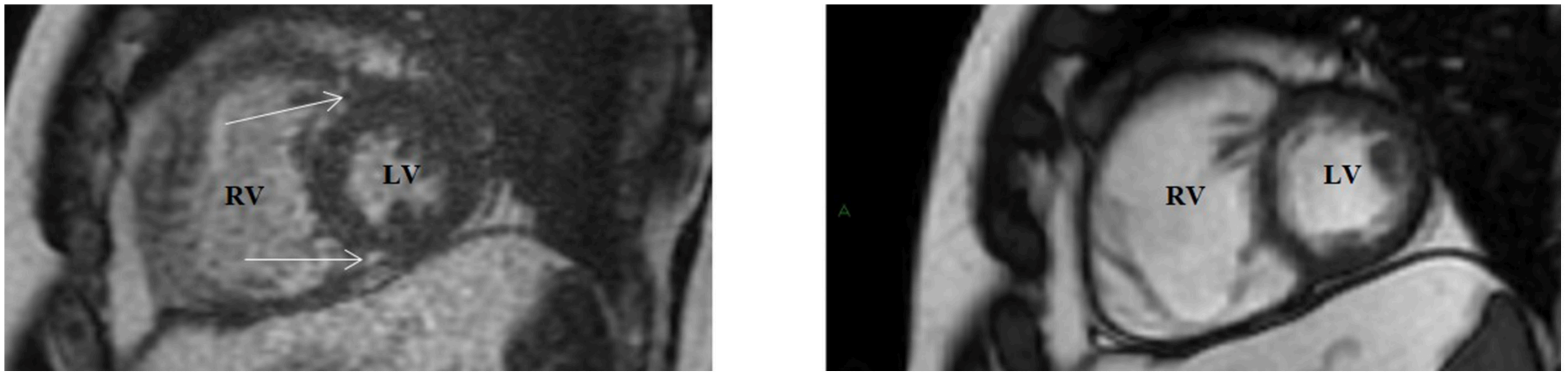
Delayed enhancement (DE) cardiac magnetic resonance in the short-axis view **(Left)** showing gadolinium hyperenhancement at the right ventricular septal insertion points (arrows), with corresponding slice on cine imaging **(Right)** showing right ventricular enlargement. LV, left ventricle; RV, right ventricle.

DE-CMR (Figure 6) can identify areas of prolonged retention (i.e., delayed clearance) of GBCA from the extracellular space, which becomes increased in areas of fibrosis and scar. This can be seen as DE at the RV septal insertion sites near the base of the heart (50, 51), a frequent finding in PH. >80% of patients with PH have some amount of DE, with almost 30% having DE involving the RV insertion points and extending to the mid-myocardial wall of the IVS (52). The dynamic changes that occur in the motion of the IVS secondary to increased mechanical stress in the setting of chronically high RV pressures are hypothesized to cause fibrosis and abnormal DE at these insertion points (53, 54) and correlates with lower RVEF. PH-associated DE is correlated to increased main pulmonary artery (MPA) pressures, RV hypertrophy, and RV dilation (55), in particular DE involving the IVS is more strongly associated with other parameters of RV failure (52).

#### PC and Cine Imaging Techniques in Relation to RH-PA Flow and Hemodynamics

PC-CMR permits the assessment of shunts, forward and regurgitant flow, RVSV, and PA velocity (45, 56), as well as advanced visualization of flow patterns to evaluate PA distensibility, coronary flow reserve, wall shear stress (WSS), and turbulence (57). PC velocity mapping is a sequence that is used to measure velocity and blood flow in vessels. PA blood flow can be measured using 2D PC flow sequences (58). This quantifies flow based on magnetic field gradients inducing phase shifts in moving protons that are proportional to their velocity and the strength of the velocity encoding (VEnc) gradient that is used. PC-CMR can be prescribed such that it detects the flow either through-plane or in-plane with respect to the plane of image acquisition. For quantitative 2D PC-CMR, image acquisition is prescribed via the through-plane mode, with the most accurate quantification obtained when the imaging plane is orthogonal to the vessel of interest. Peak PASP can be derived using the modified Bernoulli equation and identifying the peak TR velocity via 2D PC-CMR at the level of the TV, analogous to the method use in Doppler TTE.

**Figure 7:**
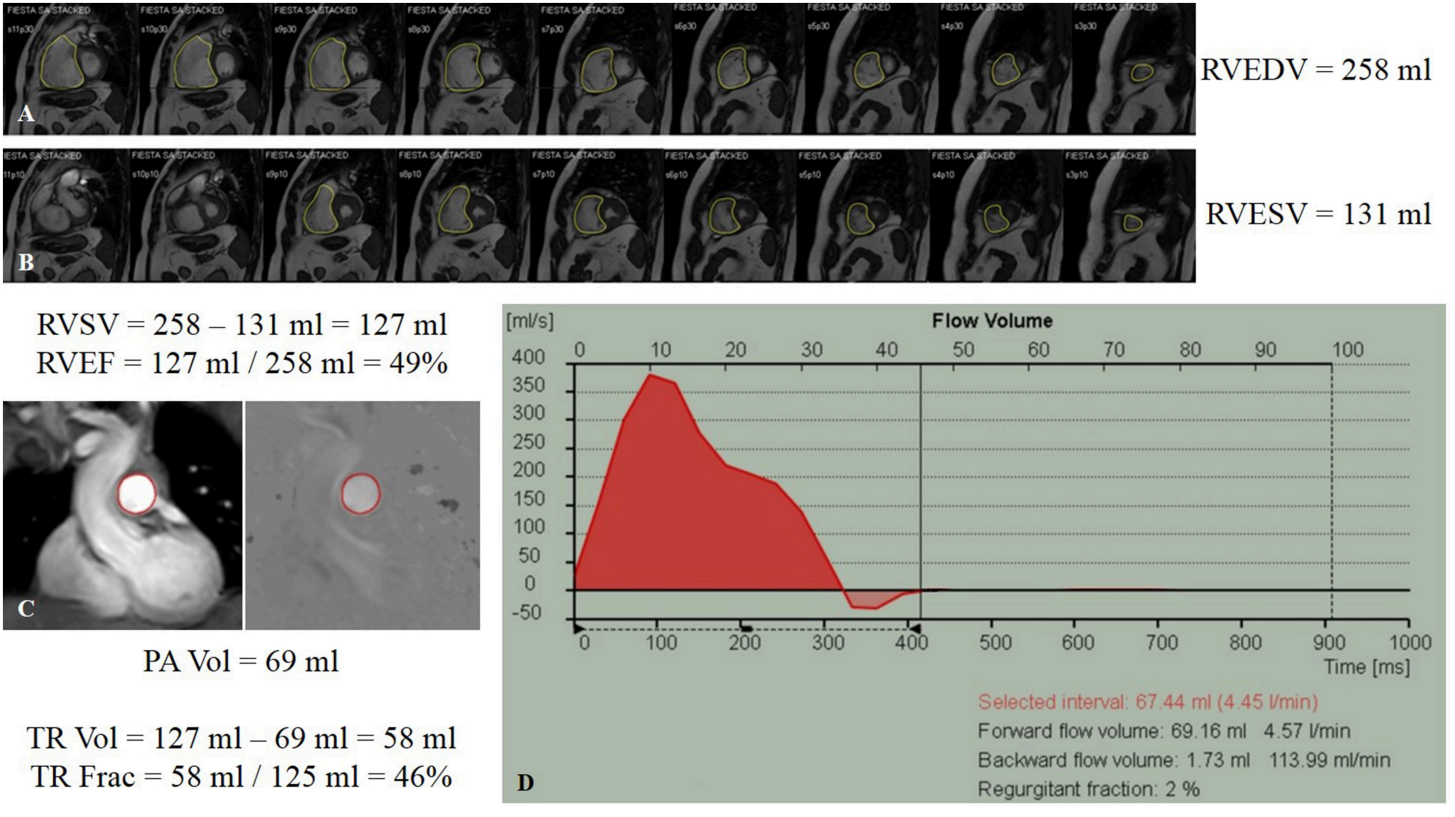
Cardiac magnetic resonance volumetric and flow analysis of a patient with group 3 pulmonary hypertension. **(A)** Steady-state free precession cine imaging stack in the short-axis view displayed from base (left) to apex (right) at end-diastole **(A)** and end-systole **(B)**. The endocardium of the right ventricle has been traced (yellow contours) with signs of septal straightening and change from a crescent contour to a more rounded contour. The slice contours have been summated, showing right ventricular end-diastolic and end-systolic volumes, and the calculated right ventricular ejection fraction. **(C)** Phase-contrast (PC) imaging performed through the proximal pulmonary artery (red contours) with magnitude- (left) and phase- (right) encoded images in corresponding spatial orientation. **(D)** Flow-time curve showing the volume detected in the pulmonary artery. Indirect determination of tricuspid regurgitation volume involving subtraction of the pulmonary artery flow volume derived from PC imaging from the calculated right ventricular stroke volume, as well as the tricuspid regurgitant fraction. Frac, fraction; PA, pulmonary artery; RVEDV, right ventricular end-diastolic volume; RVEF, right ventricular ejection fraction; RVESV, right ventricular end-systolic volume; RVSV, right ventricular stroke volume; TR, tricuspid regurgitation; Vol, volume.

In the setting of PH, quantifying cardiac shunts with the pulmonary-to-systemic flow ratio (Qp:Qs) by performing PC-CMR at the MPA and ascending aorta (AAo) (40). RVSV can be calculated by performing volumetric analysis (RV end-diastolic volume–RV end-systolic volume) or by using PC imaging to measure flow in the MPA (Figure 7). In the presence of considerable TR, RVSV calculated by MPA flow is more reliable than volumetric analysis because the latter often overestimates the true systolic volume (59). Another important parameter that can be evaluated with PC imaging is the average flow velocity in the MPA. Investigators have reported a decrease in the average MPA velocity in patients with PH; mean average velocity in MPA was <11.7 cm/s, with 93% sensitivity and 82% specificity for detecting PH when compared to RHC-derived measurements of PA pressures and resistance (60).

Distensibility is defined as the relative change in cross-sectional area (CSA) of the MPA throughout the cardiac cycle multiplied by the pulse pressure required to induce that change. Since the MPA pulse pressure is not readily known, the pulsatility or relative area change (RAC) is used as a surrogate marker of MPA stiffness (61), where RAC is calculated as:

$$\frac{\text{maximum MPA CSA}-\text{minimum MPA CSA}}{\text{maximum MPA CSA}}\;$$

MPA RAC can be combined with RHC data to calculate additional indices of proximal PA stiffness, including compliance, distensibility, elastic modulus, and stiffness index (61, 62), for which virtually all types of PH result in proximal PA stiffening and contribute to progressive decline in RV function.

PC-CMR can also evaluate coronary perfusion of the RV and detect RV ischemia, which leads to RV failure in PH (18). One study of PAH patients found that right coronary artery (RCA) peak and mean systolic flow was significantly decreased compared to control patients; these changes were inversely correlated to RV mass and RV pressure (63). Finally, PC-CMR has also been used to determine advanced hemodynamic parameters such as pulse-wave velocity, vorticity, and WSS (18). With newer, faster scanners, PC-CMR has evolved into more sophisticated acquisitions, including time-resolved, 3D, and four-dimensional (4D) flow magnetic resonance imaging (MRI) (64).

Proximal PA changes are common in PH. Pulse-wave velocity, the rate at which the systolic wave propagates through the vessels, in the central PAs is an imaging correlate regarding MPA stiffness (65, 66). Due to the short length of the MPA and low temporal resolution of MRI, the MPA pulse-wave velocity is typically estimated using the flow-area method (65, 67). 4D flow MRI has been used to assess the hemodynamic changes in the pulmonary circulation in the setting of PH, which can non-invasively measure complex 3D hemodynamic changes with full volumetric coverage of the RV and PA (48). There can be development of abnormal vortices in the MPA in PH (68, 69). Another parameter characterized with 4D flow is MPA WSS, which affects smooth muscle tone through mechanical transduction and has impact on vascular remodeling (70–72). Comparing PH patients with healthy controls, WSS was significantly lower (*p* < 0.05) in those with PH (73). 4D flow MRI has demonstrated low interobserver variability, high reproducibility, and good correlation between flow parameters obtained with 4D flow and 2D-cine PC-CMR (74, 75).

As PASP increases, RVSP can be rise above LV systolic pressure, impeding LV function because of shifting of the IVS into the LV outflow tract (76) and ventricular dyssynchrony between RV and LV (47, 77). Severe and prolonged PH can even lead to RV diastolic dysfunction, resulting in flattening or bowing of the IVS during diastole and impaired LV filling. IVS motion abnormalities such as flattening or leftward bowing of the IVS during diastole (due to chronic volume overload) and/or systole (due to chronic pressure overload) can be identified and suggest PH by correlating to elevated RH pressures. Septal bowing is indicative of PASP ≥67 mmHg, leading to impaired filling in early diastole and associated LV abnormalities of reduced LV end-diastolic volume and decreased LVSV (48, 49). Studies have reported varying positive correlations between ventricular mass index and mPAP (range 0.56–0.81) (40). CMR may also be employed to evaluate LV/RV interdependence and coupling (78).

### Cardiovascular Computed Tomography

Advantages of CT include ability to obtain images that require little scan time, excellent spatial and temporal resolution, and capability to comprehensively evaluate the cardiopulmonary structures. While CT plays less of a first-line role in the diagnosis of PH, the modality can have major impact on investigating the underlying cause of the PH, as it can visualize lung parenchyma, vasculature, and the heart. It can also assess CV changes secondary to PH, allowing for evaluation of disease severity. Different types of modern-day multidetector CT (MDCT) scans include non-contrast high-resolution CT (HRCT) and contrast-enhanced CT angiography (CTA) (26). The technology has moved beyond single-source 4-, 16-, and 64-slice CT scanners, and currently scanners with more detector rows (up to 128-, 256-, and 320-slice) and dual-source (DSCT) are becoming more widely used, which have improved the modality's spatial and temporal resolution (79). If the patient referred for CT already has a known diagnosis of PH, a non-ECG-gated non-contrast and contrast-enhanced CT can be performed for the evaluation of the PA and lung parenchyma. If there is a concern for PE and detailed assessment of CV structures is desired, an ECG-gated CTA can be performed.

#### Non-contrast CT Techniques Regarding Non-cardiac Structures

CT is indicated for the evaluation of the lungs in patients with chronic unexplained shortness of breath (20). A brief review follows regarding the utility of non-CV findings. Non-contrast and HRCT is used to assess the extent and severity of diffuse, interstitial lung disease (80) and chronic obstructive pulmonary disease (COPD) (81–83). It can also help in identifying pulmonary arteriovenous malformations (AVMs), pulmonary veno-occlusive disease (PVOD), and pulmonary capillary hemangiomatosis (PCH) (84).

Historically, the Framingham Heart Study (FHS) provided the largest cohort (*n* = 706) used to define normal PA size reference ranges (85). Investigators reviewed non-contrast chest CTs of this “healthy” cohort (non-smokers without obesity, hypertension, diabetes, CV disease, COPD, history of pulmonary embolism (PE), or heart valve surgery). The mean ± standard deviation (SD) main PA (MPA) diameter was 25.1 ± 2.8 mm, and the sex-specific 90th percentile cutoff value for MPA diameter was 28.9 mm in men and 26.9 mm in women. The diameter of the MPA was associated with increased risk for self-reported dyspnea (adjusted odds ratio 1.31, *p* = 0.02).

#### Contrast-Enhanced CTA Techniques Regarding Vasculature

CTA requires the IV administration of iodinated contrast agent to visualize the PA, and today has become the widely accepted modality of choice to diagnose acute PE, given its ability to now routinely evaluate main, lobar, segmental, and subsegmental PAs (86). However, while CTA has replaced V/Q in detecting acute PE, CTA's role in CTEPH is less well established (87) relative to V/Q when comparing to conventional pulmonary angiography. Nevertheless, a novel high-pitch ECG-synchronized computed tomographic pulmonary angiography (CTPA) protocol has been developed to allow for better contrast opacification of the PA at reduced radiation dose (88), making CTPA an increasingly more attractive option. Dilatation of the main, right, and left PAs is the most conspicuous finding on CT. Other findings of PH include calcification, tortuosity, and rapid tapering of the PAs. Persistently high PA pressures cause vascular remodeling, resulting in PA wall thickening and dilation (10). However, the differential diagnosis for a dilated PA on CTPA is broad. Etiologies include PH and its various causes, increased or turbulent blood flow (for example, left-to-right shunt: patent ductus arteriosus, atrial or ventricular septal defect), rheumatologic diseases (Behçet disease, Takayasu arteritis), connective tissue diseases (Marfan syndrome, Ehlers-Danlos syndrome, cystic medial necrosis), infections (tuberculosis, syphilis), trauma, or idiopathic (10). Caution should be used when using MPA dilatation as a criterion for PH in patients with pulmonary fibrosis, because MPA dilatation can occur in the absence of PH in patients with chronic pulmonary disease (89).

Besides FHS, other studies of normative reference ranges for PA size have been varied, given differences in demographics, medical comorbidities, ECG-gated examinations, use of IV contrast, window settings, and measuring methodology (the entire vessel diameter versus the vascular lumen) (10). On CTPA transverse images with the contrast ideally timed for opacification of the PAs, the MPA is evaluated at the level of its bifurcation, orthogonal to its long axis, and general consensus in the literature has been that a MPA diameter of at least 29 mm is potentially compatible with PH (24, 40), with the caveat that sensitivity and specificity for detecting PH vary based on the type of PH and PA diameter cutoff used. In an older study, MPA diameter ≥29 mm had a 97% positive predictive value, 89% specificity, and 87% sensitivity for the presence of PH (90). A systematic review of the sensitivity and specificity of different PA diameter cutoffs for identifying PH found an average reported cutoff of 29.5 mm (range 25.0–33.2) among 12 studies that included patients from different PH groups (10). Average sensitivity and specificity for detecting PH was 72% (range 47–87%) and 81% (range 41–100%), respectively. However, a diameter of <29 mm does not definitively exclude PH. In those with mild PH, the PA may only be slightly or borderline dilated, and findings in patients with PH can overlap with those in control subjects without PH (90, 91). In a study with subjects with mPAP >25 mmHg versus controls with mPAP <25 mmHg, it was concluded that mean MPA diameter in patients without PH is 27.5 ± 0.5 mm in men and is 25.7 ± 0.4 mm in women, with a threshold of 29.0 mm having a 52% sensitivity and a 90% specificity for the prediction of mPAP >25 mmHg (92). The diameters of segmental PAs should also be equal to those of adjacent bronchi. In the presence of a dilated MPA (≥29 mm), a segmental artery-to-bronchus diameter ratio of 1:1 or more in at least 3 lobes has a specificity of 100% for the presence of PH (90).

Several studies have suggested a strong association between the diameter ratio of MPA to ascending aorta (AAo) and PA pressure (in the absence of ectasia or ascending aortic aneurysm) (92, 93); an MPA to AAo diameter ratio greater than 1.0 was 70.8% sensitive and 76.5% specific in predicting mPAP >25 mmHg (92). FHS found that the normal mean ± SD ratio of MPA to AAo diameter was 0.77 ± 0.09, with a 90th percentile cutoff value for MPA:AAo ratio of 0.9 in both men and women (85). The MPA:AAo diameter ratio appears to be a stronger predictor of mPAP than MPA diameter alone or MPA diameter indexed to body surface area (BSA) (94). In those younger than 50 years of age, a MPA transverse diameter larger than that of the AAo on CTPA is also a sign of PH, with 96% positive predictive value and 92% specificity (95). MPA:AAo diameter ratio significantly correlated with RHC-derived mPAP (*r*^2^ = 0.45, *p* < 0.001); a composite index that utilizes (1) the MPA:AAo size ratio and (2) echo-derived RVSP incrementally enhanced PH detection (specificity 96%) when compared to using each modality alone (96). However, the confounding issue with incorporating aortic measurements in the diagnosis of PH by CT is that diseases unrelated to PH can cause dilatation of the AAo. The tradeoff was reduced sensitivity (59%), thus clinicians should be mindful that clinically significant disease may still be present despite a normal MPA:AAo size ratio in the setting of a concurrently dilated aorta.

As determined by ECG-gated CTPA for visualizing signs of PH, right PA distensibility (defined as the percentage change in CSA between diastole and systole, calculated by:

$$\frac{\text{maximum CSA}-\text{minimum CSA}}{\text{maximum CSA}}\times \;100$$

was reported to be an accurate non-invasive marker for PH, with reduced right PA distensibility having the strongest correlation to mPAP (97). The threshold for distinguishing between patients with and without PH was a distensibility of 16.5% (sensitivity 86% and specificity 96%).

Finally, anatomic manifestations of a profoundly dilated PA sometimes need to be considered, due to compression of critical mediastinal structures. Recognition of the compression of anatomic structures due to PH sequelae is crucial as early diagnosis and aggressive treatment is critical. Elevated PA pressure and PA size may result in extrinsic compression of the left main coronary artery and subsequent LV myocardial ischemia (98, 99). MPA diameter >4 cm and a MPA:AAo diameter ratio >1.21 pose higher risk of left main coronary artery compression and subsequent complications (100). While CTPA can suggest this diagnosis, ideally coronary angiography with intravascular ultrasound is needed for confirmation (98, 101). An enlarged PA can compress the left recurrent laryngeal nerve, resulting in cardiovocal (Ortner's) syndrome, characterized by vocal cord palsy and hoarseness. CT or MR of the head and neck may aid in identifying the site of the nerve compression, but definitive diagnosis requires laryngoscopy. Finally, a dilated PA can compress the tracheobronchial tree; the most common site of compression is where the left PA crosses the superior aspect of the left main bronchus (99).

#### Multislice CT Techniques Regarding Cardiac Findings

**Figure 8:**
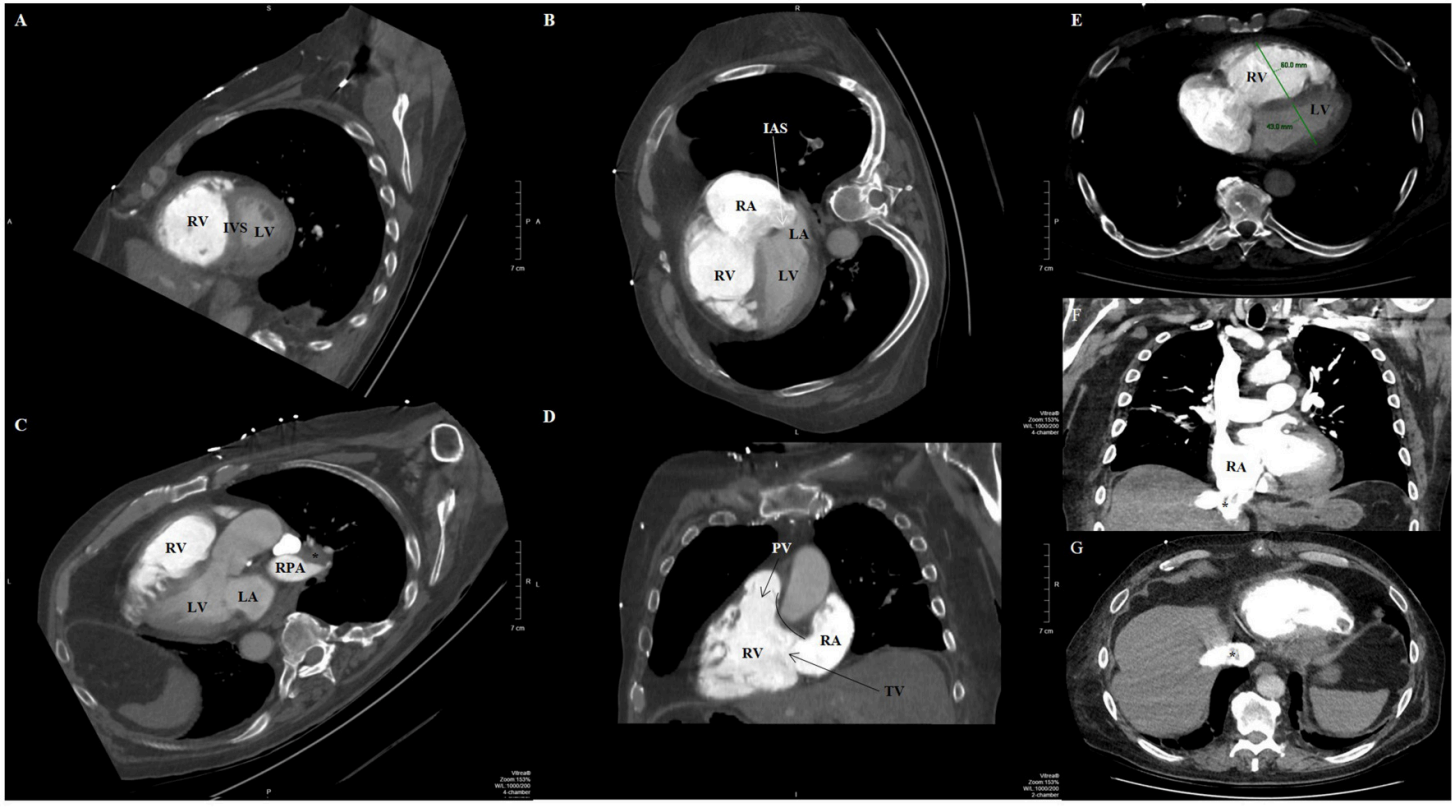
Contrast-enhanced computed tomography timed for the right heart and pulmonary artery circulation of a patient with pulmonary hypertension and pulmonary embolism, reformatted into cardiac views **(A–D)**. **(A)** Short-axis view at the level of the ventricles reveals right ventricular dilation and interventricular septal flattening and bowing toward the left ventricle. **(B)** Four-chamber view showing both right ventricular and right atrial dilatation, and bowing of the interatrial septum. **(C)** Three-chamber view showing again enlarged right ventricle, as well as filling defect (asterisk) compatible with pulmonary embolism in the right pulmonary artery. **(D)** Two-chamber view dedicated to the right heart showing the right ventricular inflow and outflow, where the tricuspid and pulmonic valves can be seen, including the discontinuity between the atrioventricular (tricuspid) and semilunar (pulmonic) valves that characterizes the morphologic right ventricle. **(E)** Transaxial view showing increased RV:LV diameter ratio (1.4). Coronal **(F)** and transaxial **(G)** views showing contrast reflux (asterisks) into the inferior vena cava and hepatic venous system compatible with significant tricuspid regurgitation. IAS, interatrial septum; IVS, interventricular septum; LA, left atrium; LV, left ventricle; PV, pulmonic valve; RA, right atrium; RPA, right pulmonary artery; RV, right ventricle; TV, tricuspid valve.

ECG gating improves evaluation of RV adaptive changes and signs of RH failure, including chamber enlargement and wall thickness hypertrophy (102). Older studies from the 1990s and 2000s correlated PH and RH dysfunction with RV hypertrophy (defined as wall thickness >4 mm), straightening or leftward bowing of the IVS, RV dilatation (defined RV:LV diameter ratio at the mid-ventricular level on axial images >1:1), RV systolic dysfunction, suggestion of TR (demonstrated by contrast reflux into the IVC and hepatic veins), dilation of IVC and hepatic veins, and/or pericardial effusion (Figure 8) (103–106). A more recent study showed that increased (≥6 mm) RV free wall end-diastolic thickness, increased RV:LV chamber lumen ratio ≥1.28, and RV:LV wall ratio ≥0.32 also predicted PH (107).

### Radionuclide Scintigraphy

Historically, prior to advent of echocardiography, radionuclide imaging was the first non-invasive modality used to obtain accurate and reproducible measurements of the RV (108). But as previously mentioned, the complex morphology of the RV with its relatively thinner wall and coarse trabeculations, as well as lack of a simple geometrical model to define its shape, has made the assessment of RV structure and function difficult. Nevertheless, strides have been made in radionuclide scintigraphy, and the RV has been assessed with the modality in certain subsets of PH. Although nuclear cardiology evaluation of the RV still suffers from several technical limitations, it can characterize systolic function, perfusion, and metabolism, providing diagnostic and prognostic information that can be integrated with data from other non-invasive imaging. Since nuclear imaging with the application of newer techniques can assess RV perfusion and metabolism in addition to morphology and EF, the modality can provide opportunities for a comprehensive RV evaluation from a single study.

#### Molecular Imaging: RV Oxygen Consumption and Perfusion/Blood Flow

With a combination of ^15^O-labeled tracers (^15^O-H_2_O, ^15^O-CO, and ^15^O-O_2_) or with ^11^C-acetate tracers, positron emission tomography (PET) imaging allows for estimation of RV myocardial oxygen consumption (109, 110). For example, resting myocardial oxygen consumption is significantly elevated in individuals with PAH (111); with progression of PAH, the efficiency of the RV increasingly decreases.

## Application of Imaging for Elucidation of Subsets of PH: Diagnostic Evaluation by Classification of the PH Patient

Initial evaluation of a patient with suspected pulmonary hypertension usually begins with an echocardiogram; however, to help define the etiology further imaging modalities are required. It is important to identify certain groups of PH for which there are specific therapies. For example, certain vasoactive substances for group 1 PAH and pulmonary embolectomy for group 4 CTEPH. Also, elucidation of group 2 or 3 PH is beneficial in order to treat the underlying left heart or pulmonary causes.

### Group 1 PH

Pulmonary arterial hypertension is usually a diagnosis of exclusion. There is not one particular imaging modality that characteristically defines PAH. In one study, left atrial volume as determined by CMR was demonstrated as having sufficient ability to distinguish idiopathic PAH from PH due to heart failure with preserved EF with 97% sensitivity and 100% specificity (112). In another study, early onset of retrograde flow in the MPA detected by standard PC-CMR quantification was characteristic of PAH (113).

Increased MPA and RV diameters and increased MPA:AAo and RV:LV ratios are correlated with increased pulmonary vascular resistance in patients with PAH in the setting of systemic sclerosis (114). However, these CT-based metrics are less predictive of PAH severity than MR imaging and echo-based measurements (115). Pulmonary AVMs in hereditary hemorrhagic telangiectasia (group 1.2.2) can be accurately detected with CT, appearing as a nodular opacity with a feeding artery and a draining vein (116, 117). While in most patients with hereditary hemorrhagic telangiectasia non-contrast CT is sufficient to identify pulmonary AVMs, CTA can be employed when characterizing larger pulmonary AVMs (118). CT findings in PVOD and PCH (group 1') reflect the underlying hemodynamic changes. With Group 1 PH, the vascular pattern on CTPA can show rapid tapering of the peripheral PAs and “corkscrew” appearance of the vessels (119). Typically with idiopathic PAH, there is symmetric enlargement of the PA.

### Group 2 PH

LV filling pressure is an important factor for defining group 2 PH due to LH disease, and this distinguishes it from group 1 PAH, in which there is no concurrent increase in LV filling pressure (120). Though echocardiography is probably the main imaging modality used in this group of patients with its ability to assess filling pressures, CMR aids in further identification, characterization, and understanding of the severity of valve or congenital heart disease. Shunts and aberrant pulmonary veins that are not easily found on echocardiography may be better seen on CMR using MRA imaging. If the echo findings are inconclusive for group 2 PH, CMR may be used to determine presence of CHD (26) or LH disease (121).

By CT, patients with PH secondary to LH disease have larger MPA diameters (severe PH, 38.3 ± 0.9 mm; mild to moderate PH, 34.9 ± 1.0 mm) than those without PH (27.4 ± 0.9 mm) (122). In addition, CTA-derived size ratio of the left to right atrium can distinguish between PH due to heart failure with preserved ejection fraction and idiopathic PAH (123). LA/RA ratio was significantly larger in those with preserved ejection fraction than idiopathic PAH (*p* < 0.001). Mean LA size was 27 ± 6 cm^2^ (transaxial view without reconstruction) and 29 ± 7 cm^2^ (after multiplanar 4-chamber view reconstruction) in those with preserved ejection fraction, compared to 19 ± 5 cm^2^ and 21 ± 5 cm^2^ for the respective views in those with idiopathic PAH (*p* < 0.001); RA size was not different between groups. The use of a size ratio provides the advantage of intrinsically accounting for patient size, and in this particular study it was demonstrated that the ratio remained unaffected by the temporal point in the cardiac cycle.

### Group 3 PH

Both non-contrast and contrast CT can accurately characterize group 3 PH. CT characterizes the type of emphysema, the presence of airway disease, and secondary features associated with smoking-related pulmonary disease (81–83, 124, 125). Recently developed quantitative CT methods of assessing emphysema severity correlate with histologic (126, 127) and clinical (128–130) parameters. Patients with PH secondary to lung disease have both increased MPA diameter and increased MPA:AAo ratio, with >1:1 being most strongly correlated with increased PASP in multivariate analysis (131).

### Group 4 PH

**Figure 9:**
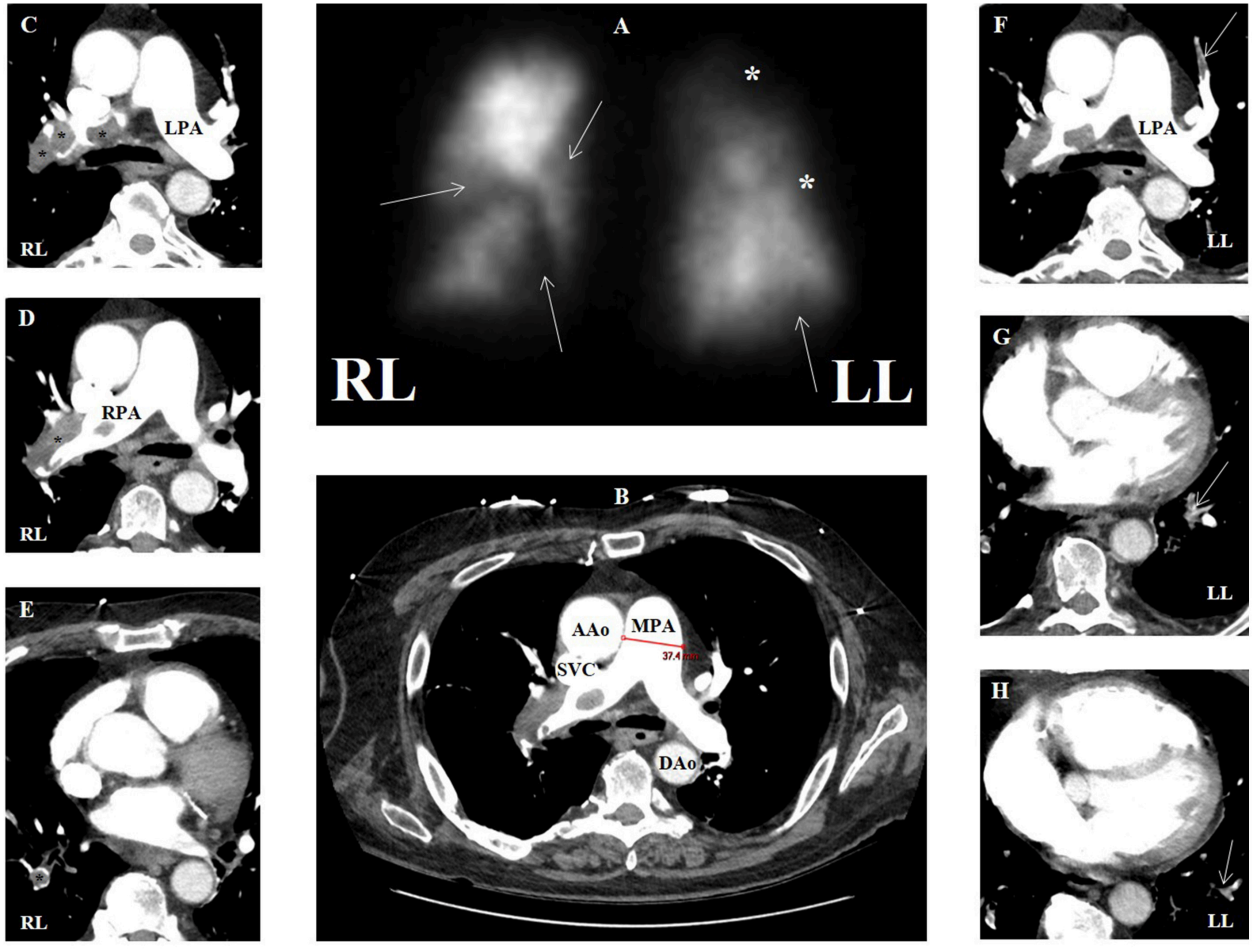
**(A)** Ventilation-perfusion scan in a patient with group 4 pulmonary hypertension with multiple segmental defects (arrows) and additional areas of possible defect (asterisks), overall with high probability for pulmonary embolism. **(B)** Contrasted computed tomographic angiography (CTA) in transaxial view of same patient showing main pulmonary artery dilation (3.7 cm) compatible with pulmonary hypertension. **(C–E)** CTA showing multiple areas of filling defect consistent with pulmonary emboli (asterisks) that correspond to perfusion defects in the right lung. **(F–H)** CTA showing multiple areas of filling defect compatible with pulmonary emboli (arrows) that may correspond to perfusion defects in the left lung. AAo, ascending aorta; DAo, descending aorta; LL, left lung; LPA, left pulmonary artery; MPA, main pulmonary artery; RL, right lung; RPA, right pulmonary artery; SVC, superior vena cava.

Though echocardiography may delineate effects of elevated right-sided pressures in patients post-PE with right ventricular hypertrophy and systolic dysfunction, and dilatation of the right chambers, evidence of vascular consequences in the evaluation of CTEPH (group 4) is gained by a ventilation/perfusion (V/Q) scan (Figure 9). CTEPH occurs eventually in up to approximately 4% of patients following an initial acute PE (132), and is more common in patients with multiple thromboembolic events (133). V/Q scanning has high sensitivity (>96%) and specificity (>90%) (87, 134) when interpreted using the modified Prospective Investigation of Pulmonary Embolism Diagnosis (PIOPED) criteria. V/Q is most useful as a screening study for CTEPH, which can be essentially excluded with a sensitivity (90–100%) and specificity (94–100%) approaching unity if the V/Q scan is normal or of low probability (135), compared to a sensitivity of 50–98% with similar specificity via CTPA (87). V/Q scan also has a higher sensitivity compared to CTPA in detecting distal areas of disease (136).

Patients with CTEPH show wedge-shaped perfusion defects with normal ventilation on V/Q scan. However, complete lack of perfusion to a lung may indicate other conditions such as malignancy, vasculitis, or fibrosing mediastinitis (136). Unmatched perfusion defects can be seen in other pulmonary vascular diseases like group 1' (PVOD and PCH), which is relevant because such patients with these conditions can develop pulmonary edema if treated with pulmonary vasodilator agents (137).

V/Q scans do not require iodinated contrast and have less radiation exposure than CTPA; the estimated effective dose for V/Q scan is 0.6–3 milliSieverts (mSv), whereas for 16-row or greater multidetector CT, it is 8–20 mSv (138). Several limitations of this imaging modality include the risk of non-diagnostic or indeterminate scans, underestimation of the extent of central vascular obstruction, and the challenge of differentiating between similarly presenting pulmonary diseases such as PVOD or fibrosing mediastinitis, resulting in increased downstream evaluation with cross-sectional imaging to confirm vascular and lung findings and to provide further information of the anatomic extent of disease (139, 140).

Group 4 CT abnormalities compatible with CTEPH are present in approximately 90% of such patients; these include RV hypertrophy, IVS straightening, complete PA luminal obstruction, intimal irregularities, pouch-like defects, bands, webs, mural thrombus, enlargement of bronchial arteries due to collateral blood supply, and mosaic lung attenuation (141, 142). With CTEPH, the vascular pattern on CTA can show more irregular PA dilation with thrombi, which may appear calcified. MPA diameter, RV:LV diameter ratio, and RV wall thickness have been found to correlate with mPAP in CTEPH (143). Although the Qanadli (144) and Mastora (145) indices have been developed to quantify the overall obstructive burden of chronic PE in these patients, these CTA parameters of PA obstruction do not correlate with RV function or pulmonary vascular resistance indices (143, 146).

Contrasted MRA of the PA showing filling defects might suggest group 4 (CTEPH) (121). Newer, dynamic contrast-enhanced (DCE) MRA is highly sensitive and specific for the diagnosis of CTEPH (147).

## Prognostication

Beyond baseline diagnosis, the various modalities offer potential prognostic information. While CXR has limited prognostic utility, chronic pericardial effusion associated with advanced PH can be seen on CXR as an enlarged cardiac silhouette described as a “waterflask” contour and is a sign of poor prognosis (104). The remaining modalities are discussed below; Table 2 highlights prognostic variables with cutoffs for negative indicators in PH.

**Table 2:** Prognostic parameters in pulmonary hypertension.

	**Threshold**	**Comment**	**References**
**ECHO VARIABLE**			
TAPSE	<18 mm	Estimate of significant RV systolic dysfunction and predictor of prognosis	(148)
RA area	≥27 cm^2^	Predictor of mortality or need for heart transplant	(149)
Diastolic eccentricity index (degree of septal shift)	abnormal index >median	Composite predictor of mortality or need for heart transplant	(150)
Pericardial effusion	present		
Indexed RA area to height	enlarged size >median		
Pulmonary vascular capacitance (stroke volume/pulse pressure)	<3.0 ml/mmHg decrease	Predictor of poor prognosis	(151)
2-dimensional strain of the basal segment of the RV free wall	<10% systolic longitudinal deformation	Predictor of poor prognosis	(152)
**MRI VARIABLE**			
Indexed RV end-diastolic volume to BSA	≥84 ml/m2	Predictors of treatment failure and mortality	(45)
Indexed stroke volume to BSA	≤25 ml/m2		
RVEF	<35%	Predictor of poor outcome, irrespective of any changes in pulmonary vascular resistance	(153)
Ventricular mass index (end-diastolic mass of RV:LV)	≥0.7	Predictor of decreased 2-year survival	(46)
RV Insertion site delayed enhancement	present	Predictor of poor prognosis	(154)
Pulmonary artery relative area change	≤16%	Predictor of mortality	(155)

*BSA, body surface area; EF, ejection fraction; MRI, magnetic resonance imaging; RA, right atrial; RV, right ventricular; TAPSE, tricuspid annular plane systolic excursion*.

### Echo Predictors of Outcome in PH

Echo is a key means of assessment of PA pressure and cardiac function at baseline diagnosis and for providing prognostic information (156). Multiple independent baseline echo variables, many reflecting RV function have been shown to be associated with survival (157). The most useful echo parameters used to determine prognosis of PH patients include RA area (149, 150), pericardial effusion (8, 150), eccentricity index (150), and TAPSE (148).

RA area is closely correlated to the degree of RAP. PAH patients who have RA area ≥27 cm^2^ are at higher risk of mortality or the need for heart transplant than those with a smaller RA area (<27 cm^2^, *p* < 0.001) (149). The 2015 ESC/ERS guidelines recommend that RA area be used to stratify patients with PAH who are at risk of clinical worsening or death (26); RA area <18 cm^2^ is suggested to confer low risk (<5%), 18–26 cm^2^ intermediate risk (5–10%), and >26 cm^2^ high risk (>10%). In an outcomes study of patients with advanced, severe PH, the prognostic significance of several echo abnormalities was described (150). RA area indexed to BSA was shown to be an independent predictor of mortality (*p* = 0.005) among PAH patients with WHO FC III-IV. Pericardial effusion was also an independent predictor of mortality (*p* = 0.003). Serial TTE can measure pericardial effusion during follow-up that when persistent can be predictive of poorer outcomes (158). Finally, abnormal diastolic eccentricity index (degree of septal shift) and increased RA size indexed to height that were greater than the median in the studied group, in conjunction with presence of pericardial effusion was shown to predict death and/or need for transplantation (150).

RV function is important because it predicts survival (1, 159, 160). TAPSE provides information about the status of RV systolic function based on the longitudinal function of RV myocardial fibers (157). In a prospective study (148), 63 PH patients underwent RHC followed by TTE to determine the prognostic significance of TAPSE. TAPSE <1.8 cm (compared to that of ≥1.8 cm) was seen in patients who had more severe RV systolic dysfunction, right heart remodeling, and disproportionate RV size relative to the LV; these patients with TAPSE <1.8 cm had significantly lower rates of survival. In PAH patients with TAPSE ≥1.8 cm, 1- and 2-year survival estimates were 94 and 88%, respectively, compared to 60 and 50%, respectively, when TAPSE <1.8 cm. TAPSE thus can predict risk of severe disease and death in PAH. Additionally, both low pulmonary vascular capacitance (stroke volume/pulse pressure) (151) and 2D strain of the basal RV free wall (152) have been shown to be predictors of poor prognosis.

### Prognostic CMR Parameters and Predictors of Outcome in PH

CMR can be used to estimate prognosis in PH in conjunction with disease severity assessment (42, 161). CMR markers that have prognostic value include RV mass, RV volume, and RV function. Increased RV end-diastolic and end-systolic volumes and decreased RVEF indicate the changes that are associated with worse prognosis in patients with PH, despite optimized medical therapy.

Increased RV end-diastolic volume has been suggested as the most precise marker for progressive RV failure (162), and reduced RVSV on CMR has been associated with increased mortality (18); CMR-derived RVSV correlates with the change in 6-min walk test distance in patients with PH (*p* < 0.0001) (163). Specific cutoffs for poor prognosis include dilated indexed end-diastolic RV volume ≥84 ml/m^2^ and reduced indexed RVSV ≤25 ml/m^2^ (45).

A significant relationship has also been shown between CMR measures of RV dysfunction in PH and serum N-terminal pro-B-type natriuretic peptide (NTpro-BNP), which is released from the ventricle in response to increased pressure or volume overload, and is a highly sensitive and specific biomarker of RV systolic dysfunction (164). One threshold for poor prognosis regarding RV systolic dysfunction is RVEF ≤35% (153). Beyond RVEF ventricular wall motion such as the degree of left septal bowing can be of prognostic value (159).

Elevated ratio of RV end-diastolic mass, compared to LV end-diastolic mass ≥0.7 predicts reduced survival at 2 years (46). Additionally, increased incidence of adverse outcomes has been associated with the presence of myocardial DE, in particular at the insertion points of the RV into the septum (154).

Parameters involving the PA can be other useful measurements of prognosis. Prolonged pulmonary mean transit time and decreased pulmonary blood flow measured with DCE MR imaging are more common in patients with PH (165, 166) and associated with worse outcomes (161). For example, decreased MPA RAC to ≤16% indicates increased MPA stiffness and is associated with increased mortality (155). PA pulsatility of deformation is another useful measure obtained with CMR and can indicate increased PA stiffness and increased risk of mortality (18).

## Follow-Up and Monitoring of Treatment

### Follow-Up Echo Assessment in PH

Echo has utility during follow-up, for example on clinical worsening of PH (167). In patients with established PH, TTE is frequently used to detect the onset of RV dysfunction because of the correlation between progressive RV dysfunction and increasing morbidity and mortality (168). In addition, echo parameters allow monitoring of medical treatment and evaluation of response to therapy (169, 170) or outcomes after pulmonary endarterectomy (171). Peak TR velocity (172), TAPSE, RA area (173), and pericardial effusion (158) are clinically relevant variables that can be assessed with echo in follow-up. However, challenges to using echo as a follow-up assessment to guide therapy include difficulties with obtaining reproducible measurements and lack of consistent methods for reporting RV size and function (174).

### Follow-Up CMR Assessment in PH

CMR may overcome some of the major limitations of TTE in the assessment of the RV, as well as avoid the repeat exposure to radioactive tracers that occur with nuclear or CT imaging. The reproducibility of CMR-derived parameters of ventricular function and mass is superior to 2D echo (175) and makes CMR useful for follow-up. Due to its non-invasive nature and lack of need for ionizing radiation, CMR has become an attractive option for serial assessments of the RV to monitor response to treatment.

Since RV failure is the most common cause of death in PH patients, it has been argued that serial CMRs can help to monitor treatment response (40). There is prognostic relevance of baseline and follow-up RVEF as measured by CMR in PAH patients (153). A smaller trial (SERAPH study) evaluated the effects of PH therapy on the RV (176). The larger EURO-MR study (177) showed that CMR measurements taken at baseline and during follow-up can provide important information about response to PAH-specific therapy, where CMR measured the effects of treatment on RVEF and LVEF, indexed RVSV and indexed LV end-diastolic volume. Other studies have measured the effects of treatment on RV volumes (42). Contrast-enhanced CMR, using the extracellular contrasting agent gadopentetate dimeglumine, can be used at follow-up to detect fibrosis at the RV insertion points of patients with severe PAH (178).

Other parameters involving the right circulation and the PA can provide follow-up information. Follow-up assessment has also evaluated extent of RV-PA coupling using both CMR (for volume assessment) and RHC (for pressure assessment) (55, 179). Severe uncoupling occurs in more severe disease due to a mismatch between increasing arterial load and a failing RV (55). Non-invasive assessment of pulmonary vascular resistance is ideal in this patient population for the assessment of response to therapy and follow-up (180). PC-CMR assessment of PA distensibility has correlated to response to vasodilator therapy, with a threshold of 10% (100% sensitivity and 56% specificity) distinguishing those who responded to those who did not (181).

### CT Guidance of and Monitoring Response to Treatment

CTA can be employed to help determine the success of embolization therapy for pulmonary AVMs (118). Distinction of PVOD and PCH (presence of small, smooth septal lines, geographic and nodular (centrilobular) ground-glass opacities, enlarged lymph nodes, and pleural effusions) from other causes of PH is critical to ensuring appropriate treatment and referral for lung transplant (182, 183). PVOD diagnosis is critical, because the pharmacologic therapy for PH uses agents that dilate the PA vascular bed. These agents can in turn cause a catastrophic collapse in perfusion pressure to the LH in cases of PVOD, potentially resulting in death.

CTPA is also important in the assessment of CTEPH patients being considered for surgical candidacy (184). While V/Q scan is still considered as the initial imaging test of choice for diagnosis of CTEPH (87), once the diagnosis is made or suggested, CTPA is commonly used to assess the extent of disease (Figures 9B–H) and the secondary cardiopulmonary changes. CTPA allows identification of proximal disease and extent of thrombi, which if limited to proximal vessels these patients may be good candidates for surgery. It can also identify distal obstructions, narrowing of the PAs and their branches, and distal stenosis (185). The presence of bronchial artery collaterals also has significance in patients with CTEPH since these patients typically do well postoperatively (186).

## The Future: New, Emerging Techniques

Technology advances in recent years have created new imaging sequences in the various four modalities, allowing for closer assessment of myocardial tissue structure and function regarding motion, deformation, and metabolism. More precise visualization of the complex RV anatomy by advanced imaging techniques and accurate measurement of blood volumes and flow may help obviate the need for invasive procedures in the future (187). Table 3 highlights some of the recent advanced tools that have been developed and are being studied.

**Table 3:** New non-invasive imaging techniques for pulmonary hypertension (PH).

**Modality**	**Parameter**	**Comments**
Echo	Speckle-tracking RV strain (188)	Normal <-25%, definitely abnormal >-20%; ≥−19% is significantly associated with all-cause mortality
	Three-dimensional echo (3DE) (189)	Comparable correlation to MRI; real-time 3DE could become a time- and cost-saving alternative to MRI
	Exercise stress echo (172)	RVSP increase ≤30 mmHg with exercise suggests worse prognosis
MRI	RV Tagging strain (190)	Predictors of clinical deterioration and death in patients with PAH
	Fast strain-encoded (SENC) imaging (191)	Real-time through-plane tagging technique that allows direct measurement of regional function via one free-breathing heart beat
	RV myocardial T1 mapping (192)	Enables measurement of myocardial extracellular volume; may be useful for detecting early stages of chronic PH, prior to onset of overt, macroscopic fibrosis
Nuclear & CT	RV FDG PET (193)	Higher uptake is associated with clinical worsening of PH and can be used in hybrid imaging
	Dual-energy CT (194)	Can provide incremental pulmonary blood volume maps and be used in hybrid imaging

*CT, computed tomography; Echo, echocardiography; FDG, fluorodeoxyglucose; MRI, magnetic resonance imaging; PAH, pulmonary arterial hypertension; PET, positron emission tomography; RV, right ventricle; RVSP, right ventricular systolic pressure*.

### Echocardiography: Speckle Tracking, 3D Echo, and Stress Echo

Software packages have been created with the capability of performing 2D analysis by speckle tracking (195). Speckle tracking can measure strain (myocardial deformation) and strain rate (speed at which deformation occurs) as variables of RV myocardial injury in PH (157). As assessed by speckle tracking strain and strain rate, the extent of myocardial damage in PH can be described as normal deformation due to afterload and contractility increase, reduced deformation due to afterload increase but preserved contractility, and reduced deformation due to afterload increase and reduced contractility. It can measure regional myocardial deformation with good correlation between PH severity and the degree of RV strain reduction (157). Diminished systolic longitudinal strain reflects the severity of RV dysfunction (152). Decreased strain has been shown to be significantly associated with all-cause mortality (188). 2D strain imaging by speckle tracking is volume and load dependent, and needs further standardization, as RV assessments based on 2D imaging can show variable reproducibility (196).

Real-time 3D echo has shown promise for measuring RV size volume and systolic function (197); it is superior to 2D echo, and can more accurately evaluate RV end-diastolic volume and RVEF (156, 198). 3D echo can provide evaluation of the RV without geometric assumptions that are integral to 2D imaging (157). It has been shown that it could provide both time and cost savings compared to MRI (189). There is some evidence that 3D echo and 2D speckle-tracking echo may be better predictors of RH failure and mortality than traditional echo variables (198). However, sometimes 3D cannot be performed since the enlarged RV cannot be completely seen within one view (189). Therefore, while 3D RV echo imaging has been an active area of investigation, this has not yet translated to routine clinical usage (199).

Echo also has capability of being utilized during an exercise stress protocol, such as with bicycle. Physiological response to a moderate amount of exercise results in a decrease in pulmonary vascular resistance and modest increase in mPAP, correlating to increased cardiac output. Normal values of Doppler-derived PASP in normal individuals usually remain ≤40 mmHg during exercise (200). Exercise stress Doppler echo has been used to assess exercise- and hypoxia-induced PH (201). PASP during moderate exercise was <43 mmHg in healthy adults, while in those over 55 years of age, PASP at peak exercise could reach toward 60 mmHg (201). Although exercise pulmonary pressures are not currently included in the criteria for PH, PA pressure assessment during exercise can be considered in those with shortness of breath without a known cause who have a normal resting echo to evaluate for a pulmonary hypertensive response to exercise. Finally, loss of RV contractile reserve demonstrated in patients with established PH who do not have a pressure increase of at least 30 mmHg with exercise suggests a worse prognosis (172). The caveat is the one must keep in mind that it is technically demanding due to the time-sensitive nature of the test (156).

### MRI: Strain Tagging, Strain-Encoded Imaging, T1 Mapping, Exercise CMR, Machine Learning, and Quantitative PA Flow Analysis

While assessment of global ventricular function by traditional CMR techniques have prognostic value for future heart failure (202), they are not sensitive to regionality. There has been a push in recent years to better evaluate changes in regional performance regarding myocardial strain and torsion. Myocardial tagging by CMR can allow for measurement of regional ventricular deformation (191). Tagging tracks differences in CMR signal intensity between tagged and untagged areas of tissue, applying mathematical formulae to reconstruct a 3D image of the heart (203). Strain analysis can be useful for identifying early changes in RV function before overt reductions in global RV function and ventricular dyssynchrony manifest (204). In PAH, RV strain and strain rate have been demonstrated to be predictors of RH failure and mortality (190).

The technique of fast strain-encoded (SENC) imaging can evaluate regional biventricular strain to detect regional RV dysfunction in those with normal global RV function (191). RV strain mapping provides quantitative evaluation of regional myocardial function, increasing the sensitivity of CMR to find regional abnormalities in RV function.

T1 mapping provides measurements of intrinsic longitudinal relaxation time of tissue. The technique applied to the RV can identify diffuse myocardial pathologic processes in the setting of PH, specifically in the interstitium. Measurement of RV myocardial T1 before and after the receipt of GBCA allows for quantification of the extracellular volume. It has been shown that RV T1 mapping values correlate with pulmonary hemodynamics, RV-PA coupling, and RV performance (192), and are associated with indicators of RV dysfunction (192, 205). Increased pre-contrast T1 values at interventricular insertion points on native T1 mapping has been demonstrated in pre-capillary PH (206). RV T1 mapping has promise in the detection of diffuse myocardial fibrosis in RV failure, a crucial end result in PH, and thus RV T1 mapping may have utility in determining treatment response in PH.

Additionally, there has been some work done in the realm of exercise CMR (207), including more recently a methodology using exercise stress CMR to measure changes in MPA RAC as a surrogate of MPA stiffness, which offers the potential to better understand the mechanisms that contribute to exercise-induced PH and exercise tolerance in those with PH (67, 208). Nevertheless, logistical challenges with the availability of MRI-conditional or -compatible stress equipment and the time-sensitive nature of obtaining CMR images during and immediately after exercise remain.

Finally, new frontiers of CMR involve quantitative analysis of helicity and vorticity of PA flow in PH patients, demonstrating a strong association between helicity in the PAs and ventricle-vessel coupling (209), and machine learning of 3D RV wall motion, which is beginning to enable the prediction of outcome and survival in those newly diagnosed with PH, independent of traditional risk factors (210).

These novel imaging approaches can provide unique insight into the pathophysiology of PH, and their incremental utility should be examined in prospective research studies (including PH clinical trials) to determine their utility relative to conventional imaging approaches. However, one area of ongoing limited utility with MRI remains the assessment of lung parenchyma. While newer sequences including ultra-short echo time imaging show potential, these remain still in their early stages, and more studies are needed (211).

### CT: Dual-Energy

New-generation CT scanners are capable to perform dual-energy CT (DECT), either with two x-ray sources and two detectors (DSCT) or with one x-ray source and one detector (212). DECT provides the same vascular and lung parenchymal findings as conventional CT, including PA diameter and morphology, but with incremental perfusion analysis information of the lungs (212, 213) via iodine maps fused with nuclear scintigraphy information to create lung-vessel maps (see Nuclear-CT Hybrid Imaging). Pulmonary parenchymal morphology, vascular anatomy, and functional assessment can all potentially be obtained in one test.

Single-source DECT involves rapidly switching kilovoltage (low, around 80 kVp, and high, around 140 kVp) at the x-ray tube anode with sophisticated detectors that can simultaneously separate photons of different energies, in order to exploit the differences in iodine attenuation at these different kilovoltages. DECT allows the scanner to quantitatively characterize the properties of various tissues, such as separating materials like iodine and calcium. Using a standard CTPA scan, DECT has been used to make quantitative lung perfusion and pulmonary blood volume (PBV) maps (212, 214–216) by calculating the amount of iodine in a voxel. While these PBV maps are not exactly perfusion images as they are a measurement of iodine at one time point while the scan is being acquired, they can serve as a sufficient surrogate marker of lung perfusion (194). In CTEPH, PBV maps have been used to assess pulmonary hemodynamics and surgical candidacy; there was a trend of inverse correlation between PBV values and pulmonary vascular resistance (217). DECT can also show evidence of bronchial collateral supply, a good prognostic marker in CTEPH (218).

### Hybrid Imaging

Digital radiological imaging systems in the modern day have the capability for concurrent fusion of images across multiple modalities, forming combined studies with both high spatial and contrast resolution (219). Multiple variables can be characterized simultaneously in a single study with optimal co-registration.

#### Nuclear-CT

##### SPECT/CT

SPECT and CT have more recently become merged to integrate function and perfusion information from SPECT with anatomic information from CT. SPECT/CT can merge regional lung perfusion data with 3D images to depict PA structures with pulmonary perfusion, thus to identify segments of pulmonary hypoperfusion (220), useful in the evaluation of CTEPH (221). Another study comparing those with pre-capillary PH and healthy controls calculated a perfusion redistribution index (PRI) via SPECT/CT (222). The PRI quantified gravity-dependent shifts in regional lung perfusion as a marker of pulmonary vascular reserve. The PRI was significantly reduced in those with pre-capillary PH compared to controls; the index was associated with several prognostic factors. However, SPECT/CT may underappreciate the actual extent of vascular obstruction in CTEPH (223). Therefore, the incremental utility of SPECT/CT is still being determined, and thus comparison to more traditional techniques needs to be further investigated in order for SPECT/CT to be properly assimilated into real-world practice.

##### PET/CT

PET has higher spatial resolution than SPECT and allows for attenuation correction, yielding improved RV visualization and potential for quantifying utilization of metabolic substrates (224). In addition to abnormal RV perfusion, impairment of RV metabolism is another major determinant of RH failure in PH patients (225). In PH, ^18^F FDG accumulation, a measure of the rate of glycolysis, has been shown to be elevated in the RV free wall (226). Higher uptake is associated with clinical worsening of PH (193). It has been argued that PET can assess PH severity and clinical outcome (227), thus providing a method to evaluate treatment response (225). The combination of PET and CT imaging in a single camera system provides integrated anatomical and pathological correlation of abnormal function, perfusion, and/or metabolic processes, with the fusion of both image modalities facilitating the interpretation of each other. Attenuation maps from the CT is used to optimize attenuation correction of the PET. Nevertheless, while the feasibility of PET has been shown in patients with PH, there have been heterogeneous results and the production of high-quality research regarding the utility of measuring metabolic shift in RV dysfunction in the setting of PH is still ongoing before its implementation in routine clinical practice (228).

#### PET-MRI

The anatomical information from MRI can be combined with the functional information from PET. This allows for the simultaneous acquisition of structure, function, perfusion, tissue characterization, and flow imaging, setting up for improved understanding of the RH-PA system (229).

## Conclusion

Multimodality non-invasive imaging of the RH-PA plays a vital role in the evaluation and management of PH. The imaging techniques that are used for PH are various with strengths and limitations; with the aim of value-based decision-making, a judicious step-wise approach algorithm to utilization of imaging in PH evaluation may reduce unnecessary testing and improve the value of care.

CXR is commonly used in the initial evaluation of PH. Echo is an established, reliable, and relatively low-cost tool to initially appraise the heart in suspected PH; specifically, it can be used to evaluate for PH due to LH disease, the most common cause of PH in the developed world. V/Q scan is most useful in identifying CTEPH patients, identifying mismatched perfusion defects. Cross-sectional MRI and CT imaging contributes to etiology determination and pathophysiology characterization of PH. CT can examine the lung parenchyma and help elucidate contributing causes due to pulmonary disease and other multifactorial conditions. MRI, with its ability to provide complementary information to echo, as well as incremental value regarding tissue characterization, is becoming increasingly utilized. Radionuclide nuclear cardiology studies can be used in select situations to provide information regarding function, perfusion, and metabolism.

Early disease recognition remains one of the essential challenges for future advances in imaging. Novel imaging techniques in the various modalities, including integration across modalities via hybrid imaging continue to be developed and investigated to allow for such early disease detection, which may allow for earlier preventive and therapeutic approaches to be adopted. Currently, their exact role in the diagnostic and follow-up evaluation of PH remains unclear, and thus additional studies are needed to clarify their role. Nevertheless, the development of more advanced imaging tools will hopefully increase our understanding of the pathophysiological factors related to the RH and the vascular biology of the PA circulation that lead to RV failure in PH; advances in spatial resolution, contrast perfusion, tissue characterization, and flow analysis will allow for the continued study and subsequent optimization in refining the role of non-invasive imaging for the detection and management of PH in the future. This is with the aim of perfecting their role in making the diagnosis, as well as enhancing future clinical trials regarding the response to treatment, thus guiding the management and follow-up of PH after therapy. Then that could ultimately the change the incidence, and morbid and deadly clinical manifestations of PH.

## Author Contributions

DH collected articles and devised the manuscript in collaboration with LS. Both authors critically reviewed, revised, and approved the final manuscript.

### Conflict of Interest Statement

The authors declare that the research was conducted in the absence of any commercial or financial relationships that could be construed as a potential conflict of interest.

## Abbreviations

2D: two-dimensional
3D: three-dimensional
4D: four-dimensional
AAo: ascending aorta
AVM: arteriovenous malformation
BSA: body surface area
CHD: congenital heart disease
CMR: cardiovascular magnetic resonance imaging
COPD: chronic obstructive pulmonary disease
CSA: cross-sectional area
CTEPH: chronic thromboembolic pulmonary hypertension
CT: computed tomography
CTA: computed tomographic angiography
CTPA: computed tomographic pulmonary angiography
CV: cardiovascular
CXR: chest radiography
DCE: dynamic contrast-enhanced
DE: delayed enhancement
DECT: dual-energy computed tomography
DS: dual-source
ΔP: peak pressure gradient
ECG: electrocardiogram
EF: ejection fraction
ePLAR: echocardiographic pulmonary to left atrial ratio
FAC: fractional area change
FHS: Framingham Heart Study
GBCA: gadolinium-based contrast agent
HR: high-resolution
IV: intravenous
IVS: interventricular septum
IVC: inferior vena cava
LH: left heart
LV: left ventricle
MD: multidetector
mmHg: millimeters of mercury
MPA: main pulmonary artery
mPAP: mean pulmonary arterial pressure
MRA: magnetic resonance angiography
MRI: magnetic resonance imaging
mSv: milliSieverts
m/s: meters per second
PA: pulmonary artery
PADP: pulmonary artery diastolic pressure
PAH: pulmonary arterial hypertension
PASP: pulmonary artery systolic pressure
PBV: pulmonary blood volume
PC: phase contrast
PCH: pulmonary capillary hemangiomatosis
PE: pulmonary embolism
PET: positron emission tomography
PH: pulmonary hypertension
PRI: perfusion redistribution index
PVOD: pulmonary veno-occlusive disease
Qp:Qs: pulmonary-to-systemic flow ratio
RA: right atrium
RAC: relative area change
RAP: right atrial pressure
RCA: right coronary artery
RH: right heart
RHC: right heart catheterization
RIMP: right ventricular myocardial performance index
RV: right ventricle
RVSP: right ventricular systolic pressure
SD: standard deviation
SPECT: single-photon emission computed tomography
SV: stroke volume
S': systolic velocity of tricuspid valve annulus
SSFP: steady-state free precession
TAPSE: tricuspid annular plane systolic excursion
TR: tricuspid valve insufficiency
TRjetV: trans-tricuspid valvular regurgitant jet velocity
TTE: transthoracic echocardiography
TV: tricuspid valve
V/Q: ventilation/perfusion
WHO FC: World Health Organization functional class
WSS: wall shear stress.
